# Symposia Abstracts

**DOI:** 10.1080/24740527.2022.2088025

**Published:** 2022-08-12

**Sexual dimorphism in the neuroimmune response to pain**

Nader Ghasemlou^a^, Bradley Kerr^b^ and Vivianne Tawfik^c^

^a^Anesthesiology, Biomedical & Molecular Sciences, Queen’s University, Kingston, Ontario, Canada; ^b^Anesthesiology and Pain Medicine, University of Alberta, Edmonton, Alberta, Canada; ^c^Anesthesiology, Perioperative & Pain Medicine, Stanford University, Stanford, California, USA

**Symposium Chair**: Nader Ghasemlou, PhD, Queen’s University, Anesthesiology, Biomedical & Molecular Sciences, Kingston, Ontario, Canada, nader.ghasemlou@queensu.ca @ghasemloulab

**Symposium Abstract:** Females are disproportionately affected by chronic pain compared to males, with a higher prevalence of pain conditions including arthritis, migraine and fibromyalgia, among others. Seminal work from various laboratories has shown that differing inflammatory responses underlie some of the sexual dimorphism observed in the regulation of pain. While it is now clear that interactions between the nervous and immune systems are critical mediators of both acute and chronic pain responses, the underlying molecular and cellular mechanisms controlling these differences remain poorly understood. We will present evidence from our respective laboratories showing how sexually dimorphic responses in neuroimmunity help control pain using models of multiple sclerosis (Dr. Bradley Kerr), complex regional pain syndrome (Dr. Vivianne Tawfik), and in the baseline control of nociception (Dr. Nader Ghasemlou).

**Speaker 1**: Nader Ghasemlou, PhD, Queen’s University, Anesthesiology, Biomedical & Molecular Sciences, Kingston, Ontario, Canada, nader.ghasemlou@queensu.ca @ghasemloulab

**Speaker 1 Abstract Title**: Circadian rhythms control somatosensory function in male mice

**Speaker 1 Abstract**: Circadian (24-hour) biological rhythms are controlled by the suprachiasmatic nucleus and entrained by external cues such as light, temperature, and feeding cycles. However, most mammalian cells also express circadian genes (also called Clock genes), such as the master controller Bmal1, which act as transcription factors to regulate the rhythmic transcription of other clock-controlled genes. These regulated genes comprise 5-50% of all genes depending on the cell and organ. Evidence now suggests that peripheral sensory neurons, as well as most immune cells, express these circadian transcription factors. Recent data from our laboratory now shows that baseline thermal, but not mechanical, thresholds in naive mice fluctuate in a rhythmic pattern, in a sex-specific manner. Male but not female mice exhibit an attenuated thermal response during the dark (resting) phase relative to the light (active) phase. Acute nociception induced by capsaicin, but not mustard oil, follows a similar trajectory. Working to better understand the molecular mechanisms underlying this response, we will present evidence that these functional outcomes are controlled by the circadian master gene Bmal1. Furthermore, our results suggest that opioid receptors are key players in this response. Our work provides new understanding of how somatosensory function and nociception may be under control of circadian rhythms and the molecular mechanisms underlying this response.

**Speaker 2**: Bradley Kerr, PhD, University of Alberta, Anesthesiology and Pain Medicine, Edmonton, Alberta, Canada, bjkerr@ualberta.ca @BradleyKerr20

**Speaker 2 Abstract Title**: How does the PNS inform the CNS about pain in multiple sclerosis?

**Speaker 2 Abstract**: Multiple Sclerosis is a disease that is associated with significant demyelination of axonal tracts in the central nervous system (CNS). Demyelinating plaques in the CNS underlie the pathological signs of weakness and paralysis that are most commonly associated with the disease. However, a significant proportion of patients with MS also develop sensory disturbances including pain in the distal limbs and/or a form of facial pain called trigeminal neuralgia. In this talk I will present recent data from my laboratory implicating the peripheral nervous system, specifically the sensory neurons that reside outside of the CNS in structures called the dorsal root ganglia (DRG) and trigeminal ganglia (TG) that innervate the distal limbs and face respectively, as key drivers of pain in the MS disease state. I will discuss recent insights into maladaptive changes that occur in the DRG and TG even in the absence of overt demyelination. I will discuss how the plasticity of the sensory neurons in the peripheral nervous system affects pain sensitivity in a specific mouse model used to study MS. Sex differences in these responses will also be discussed.

**Speaker 3**: Vivianne Tawfik, MD, PhD, Stanford University, Anesthesiology, Perioperative & Pain Medicine, Stanford, California, United States, vivianne@stanford.edu, @TawfikLab

**Speaker 3 Abstract Title**: Location-, time- and sex-specific contributions of myeloid-lineage cells to pain chronification

**Speaker 3 Abstract**: Activated myeloid-lineage cells, macrophages peripherally and microglia centrally, contribute to the acute-to-chronic pain transition, however, the details on the timing and possible sex-specificity of such involvement remains a matter of debate. For example, there is evidence that CNS microglia may contribute to chronic pain only in males. In this talk I will discuss data from my laboratory using complementary pharmacologic and transgenic approaches in mice to more specifically manipulate myeloid-lineage cells using a model of the pain condition, complex regional pain syndrome. I will discuss a novel spatiotemporal transgenic mouse line, Cx3CR1-Cre ERT2 -eYFP;TLR4 fl/fl (TLR4 cKO) that we used to specifically knock out toll-like receptor 4 (TLR4), only in microglia and no other myeloid-lineage cells. Using this transgenic mouse, we find that early TLR4 cKO results in profound improvement in chronic, but not acute, allodynia in males, with a significant but less robust effect in females. In contrast, late TLR4 cKO results in partial improvement in allodynia in both sexes, suggesting that downstream cellular or molecular TLR4-independent events may have already been triggered. I will further discuss new data using a transgenic mouse that allows for microglia-specific depletion, Cx3CR1-Cre ERT2 -eYFP;iDTR lox-STOP-lox (microglia cKO). We performed microglial depletion at multiple time points after peripheral injury and see the most striking decrease in mechanical allodynia in males and females when depletion is performed several weeks after injury. Overall, we find that microglia themselves contribute to the chronic pain transition in both sexes, however, microglial TLR4 contributes more heavily to the transition in males.

**Learning Objective 1**: Describe how nociception is differentially affected by circadian rhythms in a sex-specific manner.

**Learning Objective 2**: Describe the peripheral pathways that lead to central MS pain in male and female mice.

**Learning Objective 3**: Describe how peripheral and central myeloid lineage cells contribute to chronic pain in a sexually dimorphic manner.

**The impact of another’s pain: Psychological, physiological, and neural sequelae of observing pain in loved ones**

Rebecca Pillai Riddell^a^, Shaylea Badovinac^a^, Tine Vervoort^b^ and Marina López-Solà^c^

^a^Department of Psychology, York University, Toronto, Ontario, Canada; ^b^Ghent University, Ghent, Belgium; ^c^University of Barcelona, Barcelona, Spain

**Symposium Chair**: Rebecca Pillai Riddell, PhD, York University, Department of Psychology, Toronto, Ontario, Canada, rpr@yorku.ca @drbeccapr

**Symposium Abstract:** Observing the pain of others brings about a cascade of biological and psychological changes in the self (Goubert et al., 2005, 2021; Hadjistavropoulos et al., 2011). For caregivers and loved ones of individuals in pain, the ability to adaptively manage these reactions is closely related to the ability to provide appropriate pain assessment and pain management behaviours. This three-part symposium examines the psychological, physiological, and neural responses of individuals observing their loved ones as they undergo a range of painful experiences, across the lifespan. The workshop will be introduced with a personal reflection by session chair, Rebecca Pillai Riddell, a pain scientist with lived experience in supporting a spouse with chronic pain. In the first talk, focused on the toddler period, Shaylea Badovinac will present biobehavioural research on mechanisms subsuming how physiological arousal relates to parents’ concurrent psychological distress and use of sub-optimal soothing behaviours. In the second talk, focused on parents of school-aged children, clinical psychologist Tine Vervoort will explore how experimentally manipulating parents’ attention to children’s pain impacts parents’ physiological arousal, emotional distress, and pain control behaviour during their child’s participation in a painful task. In the third talk, neuroscientist Marina López-Solà will discuss adults’ functional brain activity in reaction to watching their romantic partner receive painful heat stimulation and how this varies as a function of partners’ interpersonal closeness.

**Speaker 1**: Shaylea Badovinac, MA, York University, Department of Psychology, Toronto, Ontario, Canada, sdbadov@yorku.ca

**Speaker 1 Abstract Title**: Parents’ physiological responses during toddler vaccinations: Associations with parents’ psychological and behavioural responses

**Speaker 1 Abstract**: Current theory and research support a central role for parental caregivers in the management of young children’s acute pain-related distress. Parents’ observable responses to their children’s pain are hypothesized to reflect a complex interplay of biological and psychological factors, however, few studies have investigated the impact of infants’ pain-related distress on parents’ physiological and psychological reactivity. A better understanding of the biological and psychological context for parents’ pain management behaviours may inform strategies for identifying and supporting parents whose own distress is impeding on their ability to respond appropriately to their infants’ pain. The aim of this presentation is to characterize parents’ cardiac responses to toddlers’ acute procedural pain and explore associations with parents’ concurrent behavioural and psychological responses. Data are drawn from a cohort of parent-infant dyads followed longitudinally across routine vaccinations over the second year of life. First, individual variability in caregivers’ cardiac responses within and across time points will be discussed in relation to infant and caregiver characteristics. Next, associations among caregivers’ cardiac responses and their concurrent psychological distress and use of sub-optimal soothing behaviours in reaction to children’s pain-related distress will be presented. To conclude, findings will be interpreted in the context of current theories and existing research pertaining to parents’ physiological and psychological experiences of their child’s pain. Directions for future research and implications for pain management will also be discussed in view of the present findings.

**Speaker 2**: Tine Vervoort, PhD, Ghent University, Ghent, Belgium, Tine.Vervoort@UGent.be, @tinevervoort13

**Speaker 2 Abstract Title**: Parental emotional responses when facing their child in pain: the role of attention and perspective-taking

**Speaker 2 Abstract**: Research has shown that observers are likely to experience emotional distress when faced with another in pain, often prompting or motivating protective behaviour aimed at controlling the sufferers’ pain. Ironically, pain control behaviors (e.g., restricting the sufferers’ painful activities) can in some situations contribute to sufferers’ pain and disability. These dynamics are particularly critical in highly dependent relationships, such as that between parent and child. In this presentation, Dr. Vervoort will provide evidence showing observers’ ability to regulate pain-related distress is key to understanding links to motivational and behavioural outcomes and that observers’ attention to pain and perspective taking may comprise key components of both successful emotion regulation and goal-directed behaviour. Dr. Vervoort will discuss lab-based findings on the role of parental emotion regulation via attention deployment to child pain and perspective taking in understanding links to motivational and behavioural outcomes. Furthermore, she will also discuss how integrating research on emotional, motivational, and interpersonal dimensions of pain (including other-oriented and self-oriented goals) may propel forward theoretical and clinical intervention development. Current evidence will be critically reviewed with discussion of future empirical and clinical directions.

**Speaker 3**: Marina López-Solà, PhD, University of Barcelona, Barcelona, Spain, mlopezsola@ub.edu, @mlopezsola82

**Speaker 3 Abstract Title**: Neural underpinnings of observing loved ones’ pain: The Vicarious Pain Signature in romantic partners

**Speaker 3 Abstract**: Pain is a phenomenon consisting of biological, psychological, and social aspects. While it has long been known that the interpersonal context impacts an individual’s experience and expression of pain, only recently has the experience of those witnessing a loved one’s acute pain accrued attention. Previous literature suggests that observing the pain of strangers activates neural mechanisms that are distinct from those implicated in processing somatic pain. An understanding of how the painful experiences of familiar and significant others are processed at the neural level may help explain individual differences in caregiving reactions in a pain context. In this presentation, Dr. López-Solà will present functional neuroimaging data that characterize the neural correlates of empathy for a loved one’s acute pain. Findings are drawn from a series of laboratory-based studies that exposed healthy adults and their romantic partners to painful heat stimulation and recorded their neural responses. Dr. López-Solà will show how observing the pain of a loved one brings about a pattern of whole-brain activation, coined the Vicarious Pain Signature (VPS), which is distinct from neural patterns reflecting one’s first-person experience of pain. She will also discuss how the magnitude of VPS responding relates to relationship factors such as interpersonal closeness. Findings will be discussed in the context of the broader literature on empathic responding to the pain of strangers to illustrate how interpersonal connections shape empathic responding on a neural level. The presentation will conclude with a discussion of opportunities for future research and potential implications for pain management.

**Learning Objective 1**: To examine associations between parents’ physiological arousal and parents’ soothing behaviours during toddler immunizations.

**Learning Objective 2**: To explore interrelationships between parents’ attention to child pain, physiological and emotional reactivity, and pain-related caregiving behaviours in an experimental context.

**Learning Objective 3**: To discuss the neural networks involved in adults observing their romantic partners’ pain during an experimental paradigm.

**Supporting people with chronic pain via multidisciplinary online pain management programs**

Jennifer Stinson^a^, Brigitte Sabourin^b^ and Patricia Poulin^c^

^a^Faculty of Nursing, University of Toronto, Toronto, Ontario, Canada; ^b^Department of Clinical Health Psychology, University of Manitoba, Winnipeg, Manitoba, Canada; ^c^Department of Anesthesiology & Pain Medicine, University of Ottawa, Ottawa, Ontario, Canada

**Symposium Chair**: Brigitte Sabourin, B.A., M.B.A., PhD, University of Manitoba, Department of Clinical Health Psychology, Winnipeg, Manitoba, Canada, bsabourin@hsc.mb.ca

**Symposium Abstract:** With the increasing demands on clinical pain specialists and ever-growing waitlists in pain clinics across Canada, there is a need to find innovative ways for patients to access support in managing their pain and mental health. The internet has provided many people with access to information and treatment applications in several different areas of health, and with the COVID-19 global pandemic, there has never been a greater need to deliver care virtually. There is growing interest in online, self-directed pain management programs by both governing structures and patient populations, with the goal to deliver timely interventions to people that is both evidence-based, accessible, and easy to understand.Various options for internet-based multidisciplinary pain management are currently being created and tested in Canada for both adult and pediatric populations. The Power Over Pain portal aims to provide Canadians with rapid access to bilingual empirically-based, stepped care resources for the management of pain, mental health, and substance use across the lifespan. One of the proposed programs in this portal is the Internet-based Multidisciplinary Acceptance and Commitment Therapy (IMPACT) program for adults living with chronic pain. Results from the just completed feasibility study will be presented. Additionally, a novel online pain program for pediatric pain patients designed to meet the unique needs of this population will be described. Timely and widely accessible interventions targeting the biological and psychological implications of living with chronic pain conditions can significantly ease suffering in many individuals living with chronic pain.

**Speaker 1**: Jennifer Stinson, RN-EC, PhD, University of Toronto, Faculty of Nursing, Toronto, Ontario, Canada, jennifer.stinson@sickkids.ca

**Speaker 1 Abstract Title**: Development and implementation of the power over pain portal for youth with chronic pain

**Speaker 1 Abstract**: Dr. Stinson will discuss the creation, evaluation, and implementation of a stepped-care virtual solution [Power Over Pain (PoP) Portal] to improve equitable, timely access to evidence-based care for youth living with chronic pain (CP). PoP Portal is an online platform that includes self-assessment tools and a suite of evidenced virtual education (pain neuroscience) and cognitive behavioural therapy-based pain interventions that are applied adaptively based on participant preferences/needs. With support from CIHR, over the past 12 months, our team has: (1) surveyed all Canadian pediatric CP clinic directors (N=13/13) and healthcare providers who treat pediatric CP across the continuum of care (N=151) to understand barriers to pain care during the pandemic; (2) completed a mixed method study to explore pandemic impacts on pain experience and mental health among youth with CP (N=303), their siblings (N=244), and parents (N=233); (3) published a rapid scoping review of virtual care best practices for pediatric CP; 4) published an evidence gap map of available virtual care solutions across the stepped care continuum for youth with CP and (5) tested the usability to ensure ease of use and accessibility. This foundational work was synthesized to inform the co-design of the PoP Portal in partnership with a pan-Canadian youth CP advisory group. The PoP Portal was built using the infrastructure of the Wellness Together Canada program, created by Health Canada. The next phase of this research is to gather information on implementation and clinical impact of the PoP Portal in a diverse sample of youth with CP.

**Speaker 2**: Brigitte Sabourin, B.A., M.B.A., PhD, University of Manitoba, Department of Clinical Health Psychology, Winnipeg, Manitoba, Canada, bsabourin@hsc.mb.ca

**Speaker 2 Abstract Title**: Feasibility study: Improving access to pain interventions for adult patients with chronic pain through the IMPACT (Internet-based Multidisciplinary Acceptance and Commitment Therapy) Program

**Speaker 2 Abstract**: Background/Aim Accessing pain clinic services can be challenging, with long waitlists delaying patients from receiving any form of pain management treatment. We sought to create and evaluate the Internet-based Multidisciplinary Pain Acceptance and Commitment Therapy (IMPACT) Program to address this gap in pain services. Methods With patient partners’ input, we developed a multi-disciplinary, online, self-directed pain management program based on Acceptance and Commitment Therapy. The IMPACT program content contains multi-media and interactive components, including videos, audio recordings, and reflective questions. Some program videos consist of patient partners reflecting on their experiences with program themes (e.g., acceptance, values, committed action). The program also includes additional units related to exercise, medications, sleep, and communication and relationships. We conducted a feasibility study with people waiting for treatment at a tertiary pain management centre in Winnipeg, Canada. Participants completed baseline measures before accessing the program, and follow-ups immediately after completing the program and at 6-months post-completion. They provided feedback on the content throughout the program. Results Seventy-one people consented to participate, and 63 completed program enrollments. Average age of enrolled participants was 55 years (range 23-83); 76% identified as female. Seventeen participants have completed the full program and 27 participants have completed follow-up measures. Between 75% and 100% of participants recommended the various units they completed. Further outcome data and program feedback will be presented. Conclusion Based on study results, the IMPACT program shows promise in supporting individuals with chronic pain, including those who may not have timely access to pain specialist services.

**Speaker 3**: Patricia Poulin, PhD, University of Ottawa, Department of Anesthesiology & Pain Medicine, Ottawa, Ontario, Canada, ppoulin@toh.ca

**Speaker 3 Abstract Title**: Adaptation of Stepped Care 2.0 for chronic pain - Foundation of the Power over Pain portal for youths and adults

**Speaker 3 Abstract**: Chronic pain is one of many fields of practice struggling with long wait-times. Endorsed by the Mental Health Commission of Canada, Stepped Care 2.0 is resiliency-based approach, grounded in recovery principles, which providers access to a variety of interventions tailored to a person’s needs, preferences, and readiness for change. Over time and based on continuous outcome monitoring, the intensity of care can be stepped up or down. Stepped Care 2.0 curates interventions spanning the continuum of care and can include education, self-directed online treatment modules, peer support, group therapy, individual treatment, and specialist care. The Power Over Pain portal is informed by Stepped Care 2.0 and connected to Wellness Together Canada. It aims to provide youths and adults living in Canada with rapid access to a variety of interventions for chronic pain and associated mental health or substance use needs. This presentation will highlight programs available through the adult stream of Power over Pain, integration with provincial and national resources, plans for on-going evaluation, further development and implementation within community, primary, and tertiary care environments. We will also describe the development of the Power Over Pain collective, its role and opportunities for engagement. Together, the Power Over Pain collective and portal constitutes a key element of a rapid learning health system of for chronic pain in Canada.

**Learning Objective 1**: Explain the Power over Pain portal’s objectives and identify some of the planned activities in meeting these objectives.

**Learning Objective 2**: Compare the programs contained in the Power over Pain portal in terms of targeted patient populations and other considerations in providing helpful recommendations for chronic pain patients who might benefit from the portal.

**Learning Objective 3**: Describe the preliminary empirical evidence supporting two of the Power over Pain portal’s programs.

**Comprehensive integrated pain program-rehabilitation pain service: An innovative approach for precision medicine**

Dinesh Kumbhare^a^, Samah Hassan^a^, Wenqing He^b^ and Ryan Koh^a^

^a^Toronto Rehabilitation Institute, Toronto, Ontario, Canada; ^b^Department of Statistical and Actuarial Sciences, University of Western Ontario, London, Ontario, Canada

**Symposium Chair**: Dinesh Kumbhare, MD, PhD, Toronto Rehabilitation Institute, Toronto, Ontario, Canada, dinesh.kumbhare@uhn.ca

**Symposium Abstract:** Chronic pain is a public health concern affecting 21% of the Canadian population. Although there have been notable advances in pain medicine in recent decades, patient-reported outcomes remain disappointingly poor. Chronic pain is a subjective multifactorial condition, modulated by a myriad of biological, psychosocial, and environmental factors. As a result, there is tremendous inter-patient variability in the clinical presentation even within a single pain diagnosis, leading to marked heterogeneity in treatment response. Inter-patient variability has led to calls for individualized treatment approaches (i.e., precision medicine) to improve patients’ reported outcomes. The concept of precision medicine is based empirically on identifying patients’ distinguishing characteristics (i.e., phenotypic profiles of patients). These profiles allow a better understanding of patients’ pain experience, predict treatment responses and hence facilitate personalized treatment approaches. However, to implement precision medicine, physicians need to conduct a comprehensive pain assessment, collect, and analyze a diverse array of clinical data to identify these phenotypic profiles of patients. This process is extremely difficult, if not impossible, to accomplish in clinical settings especially in real-time. It also imposes a high cognitive load for physicians. The presenters in this symposium will address the challenges physicians face to implement precision medicine and share innovative methods that can potentially facilitate the process of identifying phenotypic profiles of patients in clinical settings.

**Speaker 1**: Samah Hassan, MD, MSc., PhD, Toronto Rehabilitation Institute, Toronto, Ontario, Canada, Samah.Hassam@uhn.ca

**Speaker 1 Abstract Title**: Comprehensive pain assessment

**Speaker 1 Abstract**: Chronic pain (CP) is one of the most challenging health problems facing the health care industry today. It affects 21% of the adult population in Canada. Chronic pain is a multifactorial condition. Multiple biological, psychological, and social factors modulate patients’ pain experience causing tremendous inter-patient variability in clinical presentations and marked heterogeneity in treatment response. Inter-patient variability has led to calls to implement precision medicine to improve patient outcomes. Precision medicine, however, requires comprehensive pain assessment to identify patients’ distinguishing characteristics (i.e., phenotypic profiles of patients). These profiles allow a better understanding of patients’ pain experience and hence facilitate personalized treatment approaches. Until now, pain assessment has relied mainly on a list of self-reported questionnaires to capture the experience of pain. While these measures are convenient and represent the gold-standard approach to assess patient-reported symptoms, questionnaires may not be sufficient to capture all modulating factors that may affect the patients’ pain experience. As a result, treatment planning is often associated with poor outcomes. Therefore, physicians need to adopt a biopsychosocial model for pain assessment in all their patients, which might be challenging to apply in clinical practice.This session will briefly discuss the challenges of adopting a comprehensive pain assessment and explain how to conduct a comprehensive pain assessment to capture all potential modulating factors that may affect patients’ pain experience.

**Speaker 2**: Wenqing He, PhD, Department of Statistical and Actuarial Sciences, University of Western Ontario, London, Ontario, Canada, whe@stats.uwo.ca

**Speaker 2 Abstract Title**: Development of a new pain database

**Speaker 2 Abstract**: Chronic pain (CP) is a multidimensional condition with biological, psychological, and social factors, interact together to modulate each patient’s unique pain experience. As a result, patients with CP often present with significant inter-patient variability even within the single pain diagnosis, leading to marked heterogeneity in treatment response. To unravel patient variability, we need to identify patient characteristics (phenotypes) that can stratify patients into groups or clusters with similar profiles. Through these identified profiles, physicians can reach a more consistent specific diagnosis and consequently plan for more personalized treatment. Databases have now become a potential tool for physicians as well as researchers to collect and restore patient characteristics, assessment results, and patient-reported outcomes after treatment. These data can then be used to identify patient phenotypes and evaluate responses to treatments. The quality of databases, however, depends mainly on the type of variables collected. So far, decisions to include variables mainly rely on the physicians’ and researchers’ interests for specific data, the burden placed on the patients, and the time/costs associated with the data collection process. Although these factors are important to consider, constructing a database should also follow a biopsychosocial model that can reflect the nature of CP. Through this model, the database can guarantee high-quality data collection of CP to guide decision-making and treatment planning. This session will briefly discuss the development of a new database that facilitates collecting a diverse array of clinical data that reflects the multidimensional nature of CP using valid and reliable tools.

**Speaker 3**: Ryan Koh, PhD, Toronto Rehabilitation Institute, Toronto, Ontario, Canada, ryan.koh@uhn.ca

**Speaker 3 Abstract Title**: Application of machine learning techniques in precision pain medicine

**Speaker 3 Abstract**: Precision medicine is based on algorithms that consider all modulating factors that impact patients’ pain experience to permit the establishment of personalized treatment strategies. To implement precision medicine, physicians need to process a diverse array of clinical information and weigh the influence of any potential modulating factors to identify patients’ distinguished characteristics to reach a diagnosis. Although logical and important, the process required to implement precision medicine imposes a high cognitive load for physicians since they are required to appreciate the complex interactions between a very large number of factors in real-time while they are assessing their patients.Machine learning is a method of data analysis that can be used to better understand the structure of data and fit this data into models that can be better understood and utilized by physicians. Applications of machine learning can include assessing data and identifing patient profiles or patterns, creating a clinical decision support system by understanding which variables or groups of variables are most important, predicting patient’s responses to treatments or simply aiding the physician in reaching a clinical diagnosis.This session will describe different machine learning techniques and their potential uses in the processing and analysis of the collected data to recognize patterns of specific chronic pain types to guide the implementation of precision medicine in the clinical setting.

**Learning Objective 1**: Explain how to conduct a comprehensive pain assessment to capture all potential modulating factors that may affect patients’ pain experience.

**Learning Objective 2**: Describe the development of a new database that facilitates collecting a diverse array of clinical data that reflects the multidimensional nature of chronic pain using valid and reliable tools.

**Learning Objective 3**: Describe the integration of machine learning techniques in the processing and analysis of the collected data to recognize patterns of specific chronic pain types to guide safe and effective treatment.

**Chronic pain in Canada – The trajectory of pain as a national health priority and Health Canada’s response to the Canadian Pain Task Force’s Action Plan**

Fiona Campbell^a^, Maria Hudspith^b^, Linda Wilhelm^c^, Jean-Francois Leroux^d^ and Jennifer Novak^d^

^a^University of Toronto, Toronto, Ontario, Canada; ^b^Pain BC, Vancouver, British Columbia, Canada; ^c^Canadian Arthritis Patient Alliance, Midland, Kings County, New Brunswick, Canada; ^d^Health Canada, Ottawa, Ontario, Canada

**Symposium Chair**: Fiona Campbell, MD, University of Toronto, Toronto, Ontario, Canada, fiona.campbell@sickkids.ca

**Symposium Abstract:** In September 2018, the Minister of Health directed Health Canada to establish a Canadian Pain Task Force to help the Government of Canada better understand and address the needs of Canadians who live with chronic pain. Between March 2019 and May 2021, the Task Force reviewed the literature and conducted national consultations, which led to the publications of three reports that provided an overview of the gaps, challenges and opportunities towards an improved approach to the prevention and management of chronic pain in Canada. The Task Force’s final report entitled Action Plan for Pain in Canada and released in May 2021, provides a series of recommendations towards specific and targeted actions to prevent pain, improve health outcomes for people living with chronic pain, and to address its impacts on families, communities and society. This session will involve members of the Canadian Pain Task Force and a representative from Health Canada to present the trajectory of the Task Force and provide a deeper dive into the recommendations included in the Task Force’s final report. The session also intends to provide an update on Health Canada’s response and discuss future actions supporting the implementation of the Task Force’s Action Plan.

**Learning Objective 1**: At the end of the session, participants will be able to communicate the work of the Canadian Pain Task Force and its proposed Action Plan.

**Learning Objective 2**: At the end of the session, participants will be able to identify the evidence and the process that led to the Task Force’s proposed Action Plan.

**Learning Objective 3**: At the end of the session, participants will be able to communicate how Health Canada intends to move forward on the implementation of the Task Force’s Action Plan.

**Biopsychosocial drivers and consequences of musculoskeletal pain with reflection from a patient’s perspective**

Laura Stone^a^, Massieh Moayedi^b^ and Richard Hovey^c^

^a^Anesthesiology, University of Minnesota, Minneapolis, Minnesota, USA; ^b^Faculty of Dentistry, University of Toronto, Toronto, Ontario, Canada; ^c^Faculty of Dentistry, McGill University, Montreal, Quebec, Canada

**Symposium Chair**: Laura Stone, PhD, University of Minnesota, Anesthesiology, Minneapolis, Minnesota, United States, stone@umn.edu, laurasstone

**Symposium Abstract:** The biopsychosocial model of chronic pain suggests that psychological and social factors must be considered in addition to biological factors to understand an individuals’ pain experience. It follows that these factors should also be considered to develop the most effective treatment plans. In this symposia, Dr. Stone will begin with a brief summary of the current biological understanding of musculoskeletal pain with a focus on fresh insights from human and animal studies on the role of epigenetic drivers in chronic low back pain. Since epigenetic modifications are potentially reversible, the therapeutic implications will be emphasized. Dr. Massieh Moayedi will present an overview of the psychological factors that contribute to chronic pain and will present new data on the interaction between pain and cognition, and how these could potentially be leveraged as novel therapeutic targets. Dr. Richard Hovey will focus on social factors contributing to increased suffering and will share his innovative model for a community social support network that return the patient to a person.

**Speaker 1**: Laura Stone, PhD, University of Minnesota, Anesthesiology, Minneapolis, Minnesota, United States, stone@umn.edu, @laurasstone

**Speaker 1 Abstract Title**: Biological drivers and consequences of musculoskeletal pain

**Speaker 1 Abstract**: Dr. Stone will present new insights into the biological drivers of chronic back pain with emphasis on the role of epigenetics. Since epigenetics modifications are potentially reversible, the potential therapeutic implications will be emphasized. Data from animal models demonstrating the disease-modifying effects of physical activity will be highlighted. At the end of the presentation, attendees will have an increased understanding of chronic LBP pathology, the role of epigenetics in LBP and the benefits of harnessing lifestyle change to reduce the global burden of back pain.

**Speaker 2**: Massieh Moayedi, PhD, University of Toronto, Faculty of Dentistry, Toronto, Ontario, Canada, m.moayedi@utoronto.ca

**Speaker 2 Abstract Title**: Psychological drivers and consequences of musculoskeletal pain

**Speaker 2 Abstract**: Pain is the largest health-related burden on society, and interferes with cognitive processes, resulting in forgetfulness, an inability to focus, and difficulties in abstract thinking, problem solving and decision-making. A fundamental gap in our understanding of pain is the mechanism of this interference, which would serve as a therapeutic target for pain. Prevailing models of this interference rely on distraction or salience competition, but these do not adequately fit behavioural data. Our imaging studies in chronic pain reveal a potentially different mechanism for this interaction. Specifically, the frontal polar cortex has abnormal structure and function in chronic pain. The frontal pole is implicated in cognitive branching – the ability to select a task based on its perceived value while tracking the perceived value of a competing task. Based on these findings, we propose that pain competition is not distraction or salience based, but is value-based. We show novel behavioural data supporting this concept: painful stimuli, but not iso-salient, unpleasant somatosensory stimuli, adversely affect task performance on low-value task, but not a high value task. In sum, we provide evidence for a value-based model of pain-cognition interactions in the human brain.

**Speaker 3**: Richard Hovey, PhD, McGill University, Faculty of Dentistry, Montreal, Quebec, Canada, richard.hovey@mcgill.ca

**Speaker 3 Abstract Title**: Social drivers and consequences of musculoskeletal pain with reflection from a patient’s perspective

**Speaker 3 Abstract**: Dr. Hovey has defined expertise in bridging gaps between theory and practice in areas such as patient centered care and communication in healthcare. His research approach utilizes philosophical hermeneutics in strengthening our understanding of the experiences of vulnerable and underserved populations, like those living with chronic pain and illness, cancer, disability, or the effects of medically induced trauma. He also brings the perspective as a person who has lived in chronic pain for 10 years.

**Learning Objective 1**: Integrate new knowledge on the molecular drivers of low back pain.

**Learning Objective 2**: Explain how pain competes for resources, and novel potential therapeutic targets for pain management.

**Learning Objective 3**: Describe the lived experience of pain from the perspective of a patient consultant.

**Improving chronic pain care with a single-entry system: Discussing the development and implementation of central referral and triage in a tertiary care setting**

Rachael Bosma^a^, Tania Di Renna^a^, Celeste Corkery^a^ and Laura Pus^a^

^a^Women’s College Hospital, Toronto Academic Pain Medicine Institute (TAPMI), Toronto, Ontario, Canada

**Symposium Chair**: Rachael Bosma, PhD, Women’s College Hospital, Toronto Academic Pain Medicine Institute (TAPMI), Toronto, Ontario, Canada, Rachael.Bosma@wchospital.ca

**Symposium Abstract:** Single-entry models consisting of a centralized referral and triage system have shown to improve wait times, reduce duplicate referrals and prevent cancelled appointments, in tertiary clinical settings. The Toronto Academic Pain Medicine Institute (TAPMI) is a comprehensive, interdisciplinary, tertiary pain program in Toronto. It is the only provincially funded program in Ontario to successfully implement a single-entry system for chronic pain care. TAPMI operates as a hub and spoke model, with common referral and triage across 5 academic pain centres. The central referral system has streamlined care across these sites, improved patient flow and enhanced the patient journey to access care. In addition, the centralized system processes roughly 6000 referrals a year, allowing TAPMI to collect and analyze patient demographic data, pain diagnoses and referring provider characteristics on an ongoing basis. This enables TAPMI to develop and improve its programming to better meet patient and referring provider needs.This symposium will (1) discuss the successful implementation of a central referral and triage model in chronic pain care, (2) outline the clinical and research benefits of the central intake system (i.e., development of appropriate interdisciplinary programming based on patient pain needs and experiences), and (3) discuss how the centralized system aligns with a broader Learning Health Systems perspective – an innovative and continuous improvement approach that uses cyclical, technical and social methods to improve systems with every patient treated. This interdisciplinary panel discussion will embed patient stories throughout to show the positive impact of TAPMI’s centralized model on patient care.

**Speaker 1**: Tania Di Renna, MD, FRCPC, Women’s College Hospital, Toronto Academic Pain Medicine Institute (TAPMI), Toronto, Ontario, Canada, tania.direnna@wchospital.ca

**Speaker 1 Abstract Title**: The successful implementation of a single-entry model to enhance chronic pain care: The TAPMI example

**Speaker 1 Abstract**: The important role of central triage in reducing wait times and improving patient access to timely care is well documented in the literature. The implementation of a single-entry model is consistently associated with a decrease in the time between patient referral and first assessment by a specialist physician or allied health professional. Duplicate referrals, which increase wait times and reduce efficiency of patient flow, have also shown to reduce after the implementation of a single-entry model. Similar success has been seen with the centralized referral and triage system at the Toronto Academic Pain Medicine Institute (TAPMI). TAPMI is a partnership of 5 pain clinics serving as an interdisciplinary hub for chronic pain care in Toronto, Ontario. The development of this centralized system has enabled a clearer pathway for patients, increased communication between pain specialists in the area, and enhanced the sharing of pain programs and resources across the sites. The goals of this talk are to (1) describe the development and implementation of the central intake and triaging system at TAPMI, and (2) discuss the benefits of this approach for patient access to resources and pain care. We will also identify how the centralized system has allowed TAPMI to collect and analyse patient specific data, which is used to improve service delivery (patient stories will be shared to demonstrate this). Lastly, we will answer questions from the audience on how other sites may replicate the single-entry model to enhance chronic pain care.

**Speaker 2**: Celeste Corkery, BSc (Physiotherapy), Women’s College Hospital, Toronto Academic Pain Medicine Institute (TAPMI), Toronto, Ontario, Canada, celeste.corkery@wchospital.ca

**Speaker 2 Abstract Title**: The clinical benefits of central referral: Program development guided by patient needs

**Speaker 2 Abstract**: The development of a centralized referral and triage system for chronic pain across five partner sites revealed the absence of services for patients suffering from pelvic pain. From 2017 to 2019, no publicly funded chronic pain programs accepting pelvic pain referrals existed in GTA, and the needs of these patients were unmet. In response to high volumes of referrals for this underserviced population, TAPMI developed an interdisciplinary Pelvic Pain Program. A four-month needs assessment during which new patients referred for CPP met with a pelvic physical therapist and a psychologist, served as the foundation for the development of a nonmedical component of this program. An innovative 8-week holistic Pelvic Pain Group co-facilitated by a pelvic physical therapist and a psychologist emerged from the common themes identified in the needs assessment. Based on the commonly described theme of isolation and stigma, a group-format offered patients the opportunity to connect with others living similar experiences. The voices of those suffering from pelvic pain are at the foundation of this group intervention, and as such, feedback from each iteration of the group is diligently collected, and the content and delivery method continues to evolve and be informed by patient needs. The objectives of this talk are to 1) demonstrate the benefits of the central referral system in the development of the Pelvic Pain Group; 2) describe the needs assessment and 3) describe the development and outline the components of a therapeutic Pelvic Pain group designed to meet the needs of this underserviced population.

**Speaker 3**: Laura Pus, MBA, Women’s College Hospital, Toronto Academic Pain Medicine Institute (TAPMI), Toronto, Ontario, Canada, laura.pus@wchospital.ca

**Speaker 3 Abstract Title**: Central referral and beyond: Bridging clinical, research and health system approaches to improve chronic pain care for the future

**Speaker 3 Abstract**: Central referral and triage at TAPMI has proven to be a valuable system that aligns itself well with a Learning Health System (LHS) approach. A LHS is an innovative and continuous improvement approach that utilizes a diverse range of methods to improve systems with every patient that is seen. LHS is the combination of a health and research system that is grounded in patient needs and perspectives, is driven by timely data and evidence, is supported by appropriate infrastructure, and is developed for rapid learning and improvement. The aim of a LHS is to facilitate and support continuous cycles of study, feedback, and practice change. This approach is ideal in the context of chronic pain, as a LHS is built on evidence-based, patient-centred care, with the goal to capture new knowledge as an important by-product of the delivery experience.Building on the discussion thus far of TAPMI’s centralized system, this session will speak to the relevance of LHS in the context of chronic pain. More specifically, we will discuss the learning health cycle of a LHS, key enablers of a LHS, and key opportunities of a LHS in chronic pain care. We will also engage the audience in a discussion on how equity fits into this approach, and how LHS may be adopted in the future to improve patient outcomes through its iterative process of data collection, analysis, and action.

**Learning Objective 1**: To discuss the successful implementation of a central referral and triage model in chronic pain care.

**Learning Objective 2**: To identify the clinical and research benefits of the central intake system.

**Learning Objective 3**: To discuss how a centralized system aligns with a broader Learning Health Systems perspective and the value of this approach for chronic pain care.

**The untold story: Family experiences of chronic pain**

Laurie Proulx^a^, Maria Pavlova^b^ and Katie Birnie^c^

^a^Canadian Arthritis Patient Alliance, Ottawa, Ontario, Canada; ^b^Department of Psychology, University of Calgary, Calgary, Alberta, Canada; ^c^Department of Anesthesiology, Perioperative and Pain Medicine, University of Calgary, Calgary, Alberta, Canada

**Symposium Chair**: Katie Birnie, PhD, University of Calgary, Department of Anesthesiology, Perioperative and Pain Medicine, Calgary, Alberta, Canada, kathryn.birnie@ucalgary.ca @katebirnie

**Symposium Abstract:** Chronic pain clusters in families and ripples through generations. Parent global mental and physical health contributes to youth pain outcomes through biopsychosocial mechanisms (e.g., genetic risks, parent cognitions about pain, social learning). Children of parents with chronic pain are at risk of poorer physical and psychological outcomes. Further, up to 50% of youth with chronic pain have a parent with chronic pain. Living and parenting with chronic pain results in significant levels of parenting stress, anxiety, and depression. Yet, research on family experiences of chronic pain, as well as ways to support both parents and children in an integrated way, is lacking. In the proposed symposium, our patient partner, Ms. Proulx, will share her lived experience of parenting with chronic pain, as well as results of a national survey on the key challenges of and recommendations to provide better support for parents living with chronic pain. Ms. Pavlova will present new findings on how parents and children discuss past experiences of past chronic pain flare-ups and how those verbal exchanges influence child outcomes (i.e., children’s pain memories). Dr. Birnie will present new data on the development and feasibility testing of a novel virtual group-based acceptance and commitment therapy (ACT) intervention for parents living with chronic pain. The panel includes a group of clinical researchers and a patient partner applying a developmentally-informed lens to the family model of chronic pain and discussing novel ways to support parents and children living with chronic pain.

**Speaker 1**: Laurie Proulx, Canadian Arthritis Patient Alliance, Ottawa, Ontario, Canada, laurieproulx@bell.net, @ProulxLaurie

**Speaker 1 Abstract Title**: The pain experiences of women+ during pregnancy & parenting: Unmet patient needs

**Speaker 1 Abstract**: People living with rheumatic and psoriatic diseases experience significant pain that affects their participation in various aspects of life, such as pregnancy and parenting. Inflammatory arthritis and psoriasis are each estimated to impact roughly one million people in Canada. The onset and diagnosis of these diseases commonly affects people in the prime of their lives and these individuals are often left with a variety of reproductive and sexual health-related concerns and challenges in addressing pain and other symptoms of these conditions.To better understand the needs of this population, the Canadian Arthritis Patient Alliance (CAPA), the Canadian Association of Psoriasis Patients (CAPP), the Canadian Psoriasis Network (CPN) and the Canadian Spondylitis Association (CSA) launched a survey to understand the experiences and insights about sexual and reproductive health and other key aspects of their lives, including managing chronic pain. Key challenges will be presented such as communication challenges with health care providers relating to pain management, dealing with fatigue and flares, how limitations affect their child(ren), and tips for accepting how their chronic condition affects their role as a parent. The presentation will identify work completed to date by patient organizations and identify key recommendations to provide better support to women+ in their role as parents such as evidence-based patient resources and access to person-centred interdisciplinary care and support.

**Speaker 2**: Maria Pavlova, MSc, University of Calgary, Department of Psychology, Calgary, Alberta, Canada, mpavlova@ucalgary.ca @mariavpavlova

**Speaker 2 Abstract Title**: Parent-child reminiscing and children’s pain memories in the context of pediatric chronic pain

**Speaker 2 Abstract**: Pediatric chronic pain is a prevalent, disabling, and costly condition that occurs in, impacts, and is affected by a broader social context (e.g., family, parents). Biased (i.e., exaggerated compared to the initial report) pain memories are another factor that has been posited to contribute to the onset and maintenance of chronic pain. However, the role, origin, and prognostic value of pain memories in the context of pediatric primary pain disorders are unclear. The existing studies on memory in the context of pediatric chronic pain utilized methods from acute pain memory research (i.e., repeated use of single-item pain scales) and focused on the accuracy of and biases in pain recall with mixed results. Parent-child reminiscing offers a unique framework to examine how memory for chronic pain is recalled, constructed, and reconstructed. Parent-child reminiscing also provides a snapshot of parent-child verbal interactions that have been argued to play a powerful role in children’s pain outcomes. Yet, most of the existing research focuses on immediate, and often experimental, pain experiences. Reminiscing provides an idiographic representation of memory for salient past pain experiences, and how these memories are communicated within the parent-child dyad. Ms. Pavlova will present the results of the first study examining parent-child reminiscing about past pain flare-ups, as well as its associations with children’s pain memories. Ms. Pavlova will also discuss new potential avenues in parent-led pediatric chronic pain interventions to improve long-term outcomes.

**Speaker 3**: Katie Birnie, PhD, University of Calgary, Department of Anesthesiology, Perioperative and Pain Medicine, Calgary, Alberta, Canada, kathryn.birnie@ucalgary.ca @katebirnie

**Speaker 3 Abstract Title**: Evidence for treating pain in the family: Piloting a virtual ACT intervention for parents with chronic pain in the context of pediatric pain care

**Speaker 3 Abstract**: Chronic pain runs in families. Intergenerational research has revealed that children of parents with chronic pain are at greater risk for pain, emotional, behavioural, and family problems. Although an estimated 50% of children with chronic pain having a parent with chronic pain, engagement of parents in pediatric pain interventions primarily focuses on parents’ responses and neglects to address parents’ own pain and mental health. This is problematic given the impact of parent chronic pain and mental health issues on parent behaviours and child pain outcomes. Long waitlists and poor access to care are barriers to addressing parent and child chronic pain concurrently across pediatric and adult health systems. This talk will present the protocol and preliminary testing of a virtual group-based Acceptance and Commitment Therapy (ACT) for parents with chronic pain who have a child with chronic pain. The group comprised four 90-minute weekly sessions over Zoom. Sessions 1-3 were modelled after other brief ACT interventions for adults with chronic pain. Session 4 focused on parenting with chronic pain. The parent group occurred alongside a five 90-minute weekly virtual group-based psychological intervention for children 10-17 years old with headaches and/or chronic abdominal pain. Six parent-child dyads participated. Parents reported mixed chronic pain with concurrent mental health concerns (e.g., anxiety, depression, PTSD). Pre- and post-group surveys and interviews assessed feasibility and preliminary effectiveness. Implications will be shared to inform design of concurrent parent and child interventions addressing chronic pain as an intergenerational health issue.

**Learning Objective 1**: Provide a patient partner perspective on parenting and living with chronic pain.

**Learning Objective 2**: Characterize parent-child reminiscing about past pain flare-ups and examine the influence of parent-child reminiscing on pediatric pain outcomes.

**Learning Objective 3**: Explore how parent chronic pain and concurrent mental health concerns can be feasibly and acceptably addressed in the context of pediatric pain care.

**New approaches to modelling clinically relevant aspects of pain using neuroimaging in healthy subjects**

Tim Salomons^a^, Lizbeth Ayoub^b^ and David Seminowicz^c^

^a^Queen’s University, Kingston, Ontario, Canada; ^b^University of Toronto, Toronto, Ontario, Canada; ^c^University of Maryland, Baltimore, Maryland, USA

**Symposium Chair**: Tim Salomons, Ph.D., Queen’s University, Psychology, Kingston, Ontario, Canada, tim.salomons@queensu.ca @head_like_egg

**Symposium Abstract:** Much of what we know about brain activity related to pain comes from studies using experimental pain in healthy subjects. Understanding pain mechanisms will thus depend on using appropriate models for the research question. In this symposium we describe how different aspects of the pain experience can be modeled and how they reflect neural processes as assessed through brain imaging (primarily functional MRI).Seminowicz will describe the use of prolonged pain models and the neuroimaging of descending pain modulatory and cognitive circuitry in these states. This work involved the use of three different prolonged pain models and brain recordings using EEG, fMRI and simultaneous EEG-fMRI.Ayoub will show work demonstrating the neural correlates of tonic orofacial pain using an ecologically valid model: the placement of an orthodontic separator between teeth. Activation of trigeminal nociceptive and descending modulatory circuits prior to stimulation were correlated with future pain ratings in healthy adults. Salomons will describe how habituation or sensitisation to repeated pain stimuli over an hour is stable across sessions, and how neural responses in both pain-evoked and resting state functional connectivity designs can predict this stable pattern of response. Overall, we will see how various circuits can be informative for predicting pain sensitivity and modulation. Following their presentations, the speakers will hold a panel discussion, integrating their work and taking audience questions. The topics discussed will include potential clinical applications of the findings and so on.

**Speaker 1**: David Seminowicz, Ph.D., University of Maryland, Baltimore, Maryland, United States, DSeminowicz@umaryland.edu,

**Speaker 1 Abstract Title**: Multimodal examination of prolonged pain

**Speaker 1 Abstract**: We have employed prolonged pain models in healthy individuals to capture the ongoing representations of the chronic pain experience. These include an oral capsaicin model that replicated some aspects of burning mouth syndrome, topical application of the capsaicin to the skin that recapitulates symptoms of peripheral neuropathic pain, and intramuscular NGF injections that causes use-dependent pain lasting for days. An EEG-based metric, peak alpha frequency, can reliably predict individual pain sensitivity across these models. With fMRI, we showed that descending pain modulatory circuits including PAG, amygdala, and parabrachial nuclei, are engaged during prolonged pain and predict pain sensitivity. Our EEG-fMRI work indicates that cognitive networks are related to fluctuations in peak alpha frequency during ongoing pain. The talk will conclude with potential translation back to chronic pain conditions.

**Speaker 2**: Lizbeth Ayoub, B.Sc, University of Toronto, Toronto, Ontario, Canada, lizbeth.ayoub@utoronto.ca

**Speaker 2 Abstract Title**: A laboratory-based model for examining tonic orofacial pain

**Speaker 2 Abstract**: Laboratory-based acute experimental pain studies have limited ecological validity. Here, we propose a common orthodontic procedure—the insertion of an elastomeric separator between teeth—as an ecologically valid model of tonic orofacial pain. In twenty-six healthy adults, we investigated whether pre-stimulus nociceptive and pain modulatory brain circuits were related to pain elicited by the separator. We found that pre-existing functional connectivity of key nodes within the trigeminal nociceptive and descending pain modulatory pathways were significantly correlated to subsequent peak pain ratings. This model allowed us to capture the neural correlates of individual differences to an ecologically valid pain model.

**Speaker 3**: Tim Salomons, Ph.D., Queen’s University, Kingston, Ontario, Canada, tim.salomons@queensu.ca @head_like_egg

**Speaker 3 Abstract Title**: Examining neural correlates of individual differences in habituation or sensitization to repeated experimental pain

**Speaker 3 Abstract**: In many neuroimaging designs, habituation or sensitization to repeated stimulation is treated as unwanted experimental noise and steps are taken to reduce this source of error variance (e.g. changing the site of stimulation). As part of a broader program of research aimed at understanding individual differences in vulnerability and resilience to chronic pain, we examined whether these patterns of habituation or sensitization were stable within individuals and, if so, what patterns of neural response were associated with these trait-like responses. In 68 healthy participants, we found that patterns of habituation or sensitization were stable across three sessions and that these responses were associated with activation of the hippocampus, striatum and other key regions during a separate session. Implications of these findings for better understanding individual differences in pain response will be discussed.

**Learning Objective 1**: Appraise current approaches to examining individual differences in pain response.

**Learning Objective 2**: Demonstrate how new models might help us examine these individual differences.

**Learning Objective 3**: Identify patterns of neural response associated with individual differences in pain response.

**Large-scale publicly available datasets provide novel insights into pathophysiology of chronic pain - focus on the UK BioBank**

Etienne Vachon-Presseau^a^, Andrey Bortsov^b^ and Luda Diatchenko^c^

^a^McGill University, Montreal, Quebec, Canada; ^b^University of North Carolina at Chapel Hill, Chapel Hill, North Carolina, USA; ^c^McGill University, Montreal, Quebec, Canada

**Symposium Chair**: Luda Diatchenko, MD PhD, McGill University, Montreal, Quebec, Canada, luda.diatchenko@mcgill.ca

**Symposium Abstract:** There was substantial progress lately in the development of integrated publicly available datasets of substantial sample size. One of such dataset is the UK BioBank. This large cohort of approximately 500,000 individuals (40 to 69 years at the time of recruitment) from several regions of the United Kingdom was designed to study the role of genomics in phenotypes and disease. The collected data include a rich variety of phenotypic and health-related information. Follow-up information is available, as well as linked health and medical records. All 500k study participants have genomic data. Large-scale phenotype-genotype studies including multiple pain manifestations provide new insights into plausible etiologic pathways to pain conditions. In this session, we will cover these applications using the latest methods and findings to demonstrate their practical importance and relevance to novel mechanism-based therapeutic approaches.

**Speaker 1**: Etienne Vachon-Presseau, PhD, McGill University, Montreal, Quebec, Canada, etienne.vachon-presseau@mcgill.ca

**Speaker 1 Abstract Title**: Revisiting the biopsychosocial framework for chronic pain using large scale datasets

**Speaker 1 Abstract**: Chronic pain conditions are highly prevalent, heterogeneous, and commonly overlapping with other pain conditions. The accessibility to larger cohorts of patients provides unprecedented opportunities to test and modernize the biopsychosocial framework, especially from the lens of a spectrum, where pain is studied from single site to overlapping conditions. Here, we leveraged the UK Biobank dataset that enrolled about 500,000 individuals (45-75 y.o.) and curated a series of 99 features assessing physical, psychological, demographic, and sociological factors and computed a risk score capable of predicting the number of coexisting pain sites in cross-sectional data as well as the spreading of chronic pain in longitudinal data. In contrast to current theories emphasizing the heterogeneity between pain conditions, our models revealed a largely common etiology for different single site chronic pain locations and overlapping pain conditions, except for age, sex, white ethnicity, and body mass index that depended on the condition of the pain. We then showed that the derived risk factors for overlapping chronic pain conditions were associated with pain related polygenic risk scores, inflammatory blood markers, and neuroimaging-based markers for chronic pain.

**Speaker 2**: Andrey Bortsov, MD/PhD, University of North Carolina at Chapel Hill, Chapel Hill, North Carolina, United States, andrey.bortsov@duke.edu,

**Speaker 2 Abstract Title**: Genome-wide analysis identifies significant contribution of brain-expressed genes in chronic but not acute back pain

**Speaker 2 Abstract**: Back pain is the leading cause of disability worldwide. Although most back pain cases are acute, 20% of acute pain patients experience chronic back pain symptoms. It is unclear whether acute and chronic pain have similar or distinct underlying genetic mechanisms. In this study we investigated the differences in genetic architecture between acute and chronic back pain, using the UK Biobank cohort for discovery and the Nord-Trøndelag Health Study (HUNT) cohort for replication. Our results indicate that genetic contribution to chronic back pain is greater than to acute back pain, and much of the heritability of chronic back pain can be traced to genes predominantly expressed in the CNS. At the pathway level, we found the enrichment for genes in the spinal cord ventral commissure morphogenesis pathway in both cohorts. We also mapped the genetic component of chronic but not acute pain states to genes differentially expressed in the brain in mouse pain models. In summary, chronic back pain is more heritable than acute back pain and is driven mostly by genes expressed in the central nervous system.

**Speaker 3**: Luda Diatchenko, MD PhD, McGill University, Montreal, Quebec, Canada, luda.diatchenko@mcgill.ca

**Speaker 3 Abstract Title**: Genome-wide analysis identifies distinct pathophysiology for chronic overlapping pain conditions via impaired axonogenesis in the brain

**Speaker 3 Abstract**: Chronic pain is often present at more than one anatomical location, leading to chronic overlapping pain conditions (COPC). Whether COPC represents a distinct pathophysiology from the occurrence of pain at only one site is unknown. Using genome-wide approaches, we compared genetic determinants of chronic single-site vs. multi-site pain in the UK Biobank. We found that different genetic signals underlie chronic single-site and multi-site pain with much stronger genetic contributions for the latter. Among 23 loci associated with multi-site pain, 9 loci replicated in the HUNT cohort, with the DCC netrin-1 receptor (DCC) as the top gene. Functional genomics identified axonogenesis in brain tissues as the major contributing pathway to chronic multi-site pain. Finally, multimodal structural brain imaging analysis showed that DCC is most strongly expressed in subcortical limbic regions and is associated with alterations in the uncinate fasciculus microstructure, suggesting that DCC-dependent axonogenesis may contribute to COPC via cortico-limbic circuits.

**Learning Objective 1**: At the end of this symposium participants should be able to describe the advantages of large publicly available datasets for studying pain phenotypes and beyond.

**Learning Objective 2**: At the end of this symposium participants should be able to explain the advantages of deriving predictive models for chronic pain in very large datasets.

**Learning Objective 3**: At the end of this symposium participants should be able to describe the variety and use of omics data publicly available to the pain researchers.

**New insights in pain management of the preterm infant**

Ruth E. Grunau^a^, Manon Ranger^b^, Mia McLean^c^ and Marsha Campbell-Yeo^d^

^a^University of British Columbia, Paediatrics, Vancouver, British Columbia, Canada; ^b^School of Nursing, University of British Columbia, Vancouver, British Columbia, Canada; ^c^Paediatrics, University of British Columbia, Vancouver, British Columbia, Canada; ^d^School of Nursing, Dalhousie University & IWK Health; Depart. Paediatrics, Psychology and Neuroscience, Halifax, Nova Scotia, Canada

**Symposium Chair**: Ruth E. Grunau, Ph.D., University of British Columbia, Paediatrics, Vancouver, British Columbia, Canada, rgrunau@bcchr.ca

**Symposium Abstract:** Early exposure to frequent invasive procedures is related to short and long-term alterations in brain development, cognition and behaviour in children born very preterm. Sweet tasting solutions, such as sucrose or glucose, are currently considered the gold standard treatment for routinely performed minor procedural pain in both full-term and prematurely born neonates. Despite evidence of the effectiveness in reducing behavioural pain response, concerns have been raised regarding the possibility of adverse effects following repeated exposure on the developing brain of neonates born 2-4 months early. In this symposium, supported by recent pre-clinical and clinical findings, as well as parent perspectives, we will explore current evidence and perspectives as to best practices for infant pain management in the neonatal intensive care unit (NICU).Dr. Sylvie Lambert, co-chair, will share her lived experience as a parent of extreme preterm twins hospitalized in the NICU. Dr. Manon Ranger will discuss the neuroinflammatory response patterns to early pain and/or sucrose in neonatal mice. Utilising observational data from three tertiary NICUs, Dr. Mia McLean will discuss relationships between early analgesia and sucrose exposure, neonatal brain, and cognitive and behavioural outcomes in children born very preterm. Dr. Marsha Campbell-Yeo will present randomized control trial evidence of biobehavioural and evoked brain activity responses to noxious stimuli in preterm neonates, which highlights the importance of including parents as active contributors to treatment for infant pain relief. Interactive discussion will focus on integrating current evidence with parent perspectives to inform strategies for parent engagement in infant pain management.

**Speaker 1**: Manon Ranger, Ph.D., University of British Columbia, School of Nursing, Vancouver, British Columbia, Canada, manon.ranger@ubc.ca @DrManonRanger

**Speaker 1 Abstract Title**: Pain and sucrose induced neuroinflammation in neonatal mice as a mechanism explaining adverse effects on brain and memory

**Speaker 1 Abstract**: Effective pain management in the neonatal intensive care unit (NICU) is crucial to help mitigate the known short/long-term negative consequences of early pain exposure in very preterm infants. Exposure to pain adversely affects neurodevelopment. Oral sucrose is administered routinely to reduce pain of minor procedures in the NICU and is recommended as standard care in international guidelines. The use of repeated oral sucrose to avert that pain may also adversely impact the developing brain. The mechanisms by which pain, and possibly sucrose, produce negative short- and long-term effects on brain structure and function remain unclear. We have established a neonatal mouse model that closely captures critical aspects of what preterm infants may experience in the NICU (e.g., skin-breaking procedures, oral sucrose treatments). Using this paradigm, we were the first to show that exposure to early pain and/or sucrose has profound long-term structural effects on the brain and memory. Inflammation triggered by repeated negative stimuli, such as pain, increases inflammatory cytokines, which in turn modify brain function. Early-life exposure to repeated sucrose, alone or in combination with pain, might also lead to an inflammatory response and immune alterations.In this symposium, we will present our most recent findings on whether early repeated exposure to pain, sucrose, or pain and sucrose affects pro- and anti-inflammatory cytokine levels and induces brain microgliosis in neonatal mice. Our results will add evidence supporting the current clinical concerns for the use of sucrose as a standard pain management practise in the very preterm population.

**Speaker 2**: Mia McLean, Ph.D., University of British Columbia, Paediatrics, Vancouver, British Columbia, Canada, Mia.McLean@bcchr.ca

**Speaker 2 Abstract Title**: Cross-site comparisons of neonatal brain health, cognitive and behavioural outcomes following exposure to early analgesics in children born very preterm

**Speaker 2 Abstract**: A major challenge in the care of preterm neonates is to find ways to reduce the prevalence and severity of neurodevelopmental problems thus optimizing wellbeing. Early exposure to neonatal pain (frequent invasive procedures) is related to short- and long-term alterations in brain development, neurodevelopment and behavior in very preterm children. The use of analgesic and sedative practice to treat procedural pain varies considerably across hospitals within Canada. Moreover, it is unclear whether medications regularly used for analgesia and sedation in the neonatal intensive care unit (NICU) may have some unanticipated harmful effects. Moreover, although oral sucrose reduces behavior responses to neonatal pain, effects of repeated use on the developing immature brain and neurodevelopment is unknown. In a multi-site cohort study across three tertiary NICUs we are examining whether: 1) relative exposure to morphine analgesia and sucrose, independent of procedural pain exposure and clinical confounders, are related to cognitive and behavioural outcomes at ages 18 and 33 months; and 2) cumulative sucrose exposure is related to neonatal brain health (as measured by the Hurst exponent). Our findings will provide evidence supporting safe analgesic practises for the treatment of pain in neonates born very preterm, considering brain development and neurodevelopmental outcomes. Ultimately, our work will help improve clinical care of neonatal pain and ensure children born very preterm thrive.

**Speaker 3**: Marsha Campbell-Yeo, Ph.D., NNP-BC, Dalhousie University & IWK Health, School of Nursing; Depart. Paediatrics, Psychology and Neuroscience, Halifax, Nova Scotia, Canada, Marsha.Campbell-Yeo@Dal.ca @DrMCampbellYeo

**Speaker 3 Abstract Title**: The influence of skin-to-skin contact on Cortical Activity during Painful procedures in preterm infants in the neonatal intensive care unit (iCAP mini)- Preliminary results

**Speaker 3 Abstract**: Despite level one evidence on the effectiveness of maternal or parent-led interventions, specifically breastfeeding or skin-to-skin contact (SSC), to reduce biobehavioural pain responses associated with repeated procedural pain, practice uptake in neonatal care remains underutilized. One reason cited for this lack of uptake relates to a dearth of study regarding pain assessment beyond biobehavioural response such as brain-based measures. Most notably, there is a lack of understanding of the effect of these interventions on a) noxious pain-related responses in the neonatal brain, (b) the efficacy of SSC when compared to sucrose on noxious pain-related brain activity, and (c) the relationship between noxious evoked brain activity and biobehavioural responses to noxious stimuli in preterm neonates.Preliminary data from our randomized control trial aimed to characterize the effect of skin-to-skin contact compared to 24% oral sucrose on noxious evoked activity in the preterm neonate brain undergoing a clinically required heel lance will be discussed. Potential differentiation between bio-behavioural pain response and noxious evoked pain response elicited by clinical heel lance and frequency of adverse events between interventions will be presented. Challenges and complexities in the electroencephalographic measurement of neonatal pain and maternal acceptability will also discussed.

**Learning Objective 1**: Describe most recent evidence on short-term effects of early repetitive pain and/or sucrose exposure on neuroinflammatory markers and cell morphology in a mouse model.

**Learning Objective 2**: Describe effects of early analgesics and pain exposure on neonatal brain and child cognition and behaviour in children born very preterm.

**Learning Objective 3**: Describe current evidence comparing administration of oral sucrose and parent-led pain-relieving interventions to better recognize biobehavioural and evoked brain responses to neonatal procedural pain and inform uptake of parent-led interventions.

**Epiregulin and EGFR interactions as critical contributors to pain processes and a new therapeutic pain target**

Loren Martin^a^, Gregory Neely^b^ and Luda Diatchenko^c^

^a^University of Toronto, Toronto, Ontario, Canada; ^b^University of Sydney, Sydney, Australia; ^c^McGill University, Montreal, Quebec, Canada

**Symposium Chair**: Luda Diatchenko, MD PhD, McGill University, Montreal, Quebec, Canada, luda.diatchenko@mcgill.ca

**Symposium Abstract:** The search for new chronic pain treatments continues to be a priority in the pain field due to the fact that many current therapeutic options carry the risks of addiction and/or other undesirable side effects. Recently, the crucial role in pain of a protein known as epidermal growth factor (EGFR) and its ligand epiregulin (EREG) has been discovered through human genetic association studies. Importantly, the contribution of this pathway to pain states is conserved in both mice and fruit flies. EGFR blockers, routinely used in the treatment of lung cancer to inhibit tumor growth, is demonstrated to be potent analgesics (comparable to morphine) in mouse models of inflammatory and chronic pain. Repurposing existing drugs to treat diseases other than those they were designed for can be advantageous, because the toxicity of these compounds is well characterized, making their subsequent development for new indications relatively quick and inexpensive. Furthermore, developing drugs targeting these pathways through modulation of EREG activity is providing a new avenue for a non-opioid therapy for chronic pain.

**Speaker 1**: Loren Martin, PhD, University of Toronto, Toronto, Ontario, Canada, lj.martin@utoronto.ca

**Speaker 1 Abstract Title**: Uncovering a role for the EGFR and epiregulin as novel pain targets

**Speaker 1 Abstract**: The EGFR belongs to the well-studied ErbB family of receptor tyrosine kinases. EGFR is activated by numerous endogenous ligands that promote cellular growth, proliferation, and tissue regeneration. In this talk, I will give a brief overview of this system and efforts we have made towards uncovering a role for EGFR and its natural ligand, epiregulin (EREG), in pain processing. We have shown that inhibition of EGFR with clinically available compounds strongly reduces nocifensive behavior in mouse models of inflammatory and chronic pain. EREG-mediated activation of EGFR enhanced nociception through a mechanism involving the PI3K/AKT/mTOR pathway, while EREG neutralizing antibodies reduce established chronic pain in mice. Moreover, EREG neutralizing antibodies may have a less severe side effect profile than EGFR antagonists providing a novel and advantageous therapeutic target for the treatment of persistent pain conditions.

**Speaker 2**: Gregory Neely, PhD, University of Sydney, Sydney, Australia, greg.neely@sydney.edu.au,

**Speaker 2 Abstract Title**: High throughput functional validation of conserved pain genes

**Speaker 2 Abstract**: A basic understanding of the core genes and systems that control nociception/pain can help us develop new strategies to treat pain disease. However, these are systems level processes that can be difficult to model in vitro. Since the primordial genetic architecture of our nociception system first evolved over 500 million years ago, high throughput genetic screening in insects can help us identify new conserved pain genes and pathways. By combining human genetics data with high throughput functional screening in fruit flies, we have identified or validated multiple new conserved pain genes. Here we will discuss these efforts, and how they helped confirm EGFR/ EREG as a core pathway controlling pain perception from insects through to humans.

**Speaker 3**: Luda Diatchenko, MD PhD, McGill University, Montreal, Quebec, Canada, luda.diatchenko@mcgill.ca

**Speaker 3 Abstract Title**: The dichotomous role of epiregulin in pain states

**Speaker 3 Abstract**: We systematically screened single-nucleotide polymorphisms (SNPs) in all gene loci belonging to EGFR-family receptors (namely, EGFR, ERBB2, ERBB3 and ERBB4) and their ligands (namely, AREG, BTC, EGF, EPGN, EREG, HBEGF, MUC4, NRG1, NRG2, NRG3, NRG4 and TGFA) for their association with reported clinical pain. We tested an association with self-reported pain intensity in patients with chronic facial pain who participated in the Orofacial Pain: Prospective Evaluation and Risk Assessment (OPPERA) cohort. We found that only epiregulin (EREG) was associated with pain. The strongest effect was observed for a minor allele at rs6836436 in EREG, which was associated with lower chronic pain intensity. However, the same allele was associated with higher facial pain intensity among cases with recent onset of facial pain. Similar trends were observed in an independent cohort of UK Biobank (UKB) where the minor allele at rs6836436 was associated with a higher number of acute pain sites but a lower number of chronic pain sites. Expression quantitative trait loci analyses established rs6836436 as a loss-of-function variant of EREG. Lastly, the dichotomous role of EREG for pain phenotypes was tested using mouse models of chronic and acute pain sensitivity.

**Learning Objective 1**: Upon completion of this session, attendees will be able to analyze the potential of EGFR and EREG as novel non-opioid pain therapeutics.

**Learning Objective 2**: Upon completion of this session, attendees will be able to describe the functional validation of human genetics data using fruit flies as a powerful approach for identifying critical pain genes and pathways like EGFR/ EREG.

**Learning Objective 3**: Upon completion of this session, attendees will be able to explain the analgesic role of EREG during the early stages of pain, and, an opposite-pronociceptive role in establishing chronic pain.

**Critical social science approaches to chronic pain and marginalization**

Perri Tutelman^a^, Therese Lane^b^, Laura Connoy^c^, Lise Dassieu^d^ and Bruce Wallace^e^

^a^PhD Candidate, Dalhousie University, Halifax, NS, Canada; ^b^Person with lived experience of chronic pain, Toronto, Canada; ^c^Western University, London, Ontario, Canada; ^d^Centre de recherche du Centre hospitalier de l’Université de Montréal (University of Montreal Hospital Research Centre), Montreal, Quebec, Canada; ^e^University of Victoria, Victoria, British Columbia, Canada

**Symposium Chair**: Fiona Webster, PhD, Western University, London, Ontario, Canada, fiona.webster@uwo.ca @FionaWebster1; Therese Lane

**Symposium Abstract:** The social sciences have long drawn attention to the role of social and public institutions in shaping people’s experience of health. Yet to date these approaches have been less well incorporated into chronic pain research. Studies in the area of chronic pain have historically been dominated by biomedical and clinical approaches that sometimes construct pain in dualistic and reductionist ways, thereby tending to problematize people living with pain, rather than attending to the complex and often violent historical, social, political, and economic conditions impacting their lives. To be sure, there is growing recognition of the association between pain and systemic and structural marginalization, including experiences of trauma and violence, yet more research is urgently needed. The Canadian Pain Task Force Report recently called for greater attention to this issue and several Canadian studies indicate that people with chronic pain are much more likely to struggle with poverty; such disparities are rooted in the social determinants of health – poverty, living and working conditions, and social exclusion. We argue that research in the area of chronic pain requires much more critical attention to how processes of marginalization shape experiences of chronic pain. This panel will, therefore, call attention to how critical social science approaches can be engaged to assist with efforts to critically interrogate not only chronic pain, but also the intersection of chronic pain with marginalization. In doing so, the focus on the individual can be transcended in favour of a focus on social systems.

**Speaker 1**: Laura Connoy, PhD, Western University, London, Ontario, Canada, lconnoy@uwo.ca @LauraConnoy

**Speaker 1 Abstract Title**: A critical sociology of chronic pain

**Speaker 1 Abstract**: Despite calls for inter disciplinarity in research, sociological approaches remain less utilized in chronic pain research. Reasons for this are multi-faceted. In this session, I reflect on the contributions of sociology to the study of chronic pain, with an emphasis on those studies adopting a critical lens. Scholars are beginning to call attention to the links between chronic pain and systemic and structural violence and inequity, and it is here that critical sociology can provide important insight by stressing the social as it pertains to chronic pain. Sociological research has the potential to contribute understandings of institutions, such as the capitalist system, late modernity, and neoliberal imperialism and also offers tools that allow for the exploration of relationships between race, gender, and class. This has the potential to uncover deeper understandings of chronic pain, for the individual as well as society. Upon review of existing sociological analyses of chronic pain, and drawing on findings from a critical ethnography of chronic pain and marginalization (COPE II) I share some of the important critical contributions emerging from this work and note future research directions as a means to further the advancement of a critical sociology of chronic pain subfield and promote interdisciplinary work in this area.

**Speaker 2**: Lise Dassieu, PhD, Centre de recherche du Centre hospitalier de l’Université de Montréal (University of Montreal Hospital Research Centre), Montreal, Quebec, Canada, lise.dassieu@umontreal.ca @LiseDassieu

**Speaker 2 Abstract Title**: Painful lives: investigating the social experience of chronic pain

**Speaker 2 Abstract**: This presentation draws on the results of three recent qualitative research studies that utilized a sociological approach to pain experiences: (1) a study of the chronic pain experience and management among marginalized people who use drugs; (2) an analysis of the opioid overdose epidemic impacts on the social relationships of people living with chronic pain; and (3) an investigation of the systemic inequities affecting people with chronic pain during the COVID-19 pandemic. This body of research converges to highlight the multiple and intertwined forms of stigma, prejudices, and discrimination associated with the daily social experience of chronic pain. This work also highlights the constant energy deployed by people with chronic pain in attempting to counter social stigma. Chronic pain is far more than an individual biomedical and psychological health condition, and social science approaches underscore the importance of addressing these issues collectively. Both healthcare and welfare policies need to engage in fostering the inclusiveness of people with chronic pain in society, especially those experiencing stigma, marginalization, and systemic barriers. In research, clinical practices, and decision-making, interdisciplinary approaches integrating social sciences as a core component are essential to creating safe and equitable conditions for all individuals living with chronic pain.

**Speaker 3**: Bruce Wallace, PhD, University of Victoria, Victoria, British Columbia, Canada, barclay@uvic.ca

**Speaker 3 Abstract Title**: Chronic pain in persons who are marginalized by social conditions

**Speaker 3 Abstract**: For people who experience social inequities and structural violence, pain and related care are inexorably linked to experiences of injustice and stigma. I present the analysis of a social science field study, conducted in partnership with a team of researchers and community agencies, whose purpose was to examine in greater depth the experiences of pain, discrimination and stigma across diverse marginalized groups. Themes on the relationship of pain and marginalization that emerged from the study will be discussed in the presentation and will include: social locations and identities; experiences of violence; trauma and related mental health issues; experiences of discrimination, stigma and dismissal; experiences of inadequate and ineffective health care; and, the impacts of these intersecting experiences. The paper will conclude with remarks on equity-oriented responses to chronic pain that would recognize pain not only as a biomedical issue but also one of social justice.

**Learning Objective 1**: Differentiate between critical social science and biomedical approaches to chronic pain and discuss how these approaches can be integrated through interdisciplinarity.

**Learning Objective 2**: Offer suggestions for how future approaches to chronic pain research can incorporate social science approaches to explore or include marginalization.

**Learning Objective 3**: Provide examples of social science approaches to chronic pain and marginalization by sharing results of several studies.

**Medical cannabis and cannabinoids for chronic pain: Current evidence and knowledge gaps**

Fiona Campbell^a^, Jason Busse^b^, Hance Clarke^a^ and John Brown^c^

^a^Anesthesiology and Pain Medicine, University of Toronto, Toronto, Ontario, Canada; ^b^Anesthesia, McMaster University, Hamilton, Ontario, Canada; ^c^Chronic Pain Centre of Excellence for Canadian Veterans, Dorchester, Ontario, Canada

**Symposium Chair**: Fiona Campbell, MD, University of Toronto, Anesthesiology and Pain Medicine, Toronto, Ontario, Canada, fiona.campbell@sickkids.ca @DrFCampbell)

**Symposium Abstract:** Medical cannabis is commonly and increasingly used by Canadians for chronic pain; however, this therapeutic option was made available in 2001 on compassionate grounds as opposed to empirical evidence showing that the benefits exceed the harms. Further, recreational cannabis was legalized in Canada in 2018. Several recent guidance documents have been published to help optimize evidence-based practice; however, they have inconsistent and conflicting recommendations. This symposium will discuss and reconcile contrasting recommendations for medical cannabis and chronic pain.Evidence informing the benefits and harms of medical cannabis for chronic pain is limited but increasingly available; however, the generalizability of findings is dependent on whether enrolled patients and products administered reflect real world practice. We will explore this issue and highlight areas for improvement.Finally, evidence alone is not sufficient for clinical decision-making. Patients’ values and preferences are critical to ensure that treatment decisions are accountable to an individual’s context. A patient partner will discuss their involvement in the development of a clinical practice guideline on cannabis for chronic pain.

**Speaker 1**: Jason Busse, DC, PhD, McMaster University, Anesthesia, Hamilton, Ontario, Canada, bussejw@mcmaster.ca @JasonWBusse

**Speaker 1 Abstract Title**: Reconciling contrasting recommendations for medical cannabis and chronic pain

**Speaker 1 Abstract**: Medical cannabis is increasingly used to manage chronic pain and, as of March 2020, Health Canada reported 329,000 Canadians with authorization to access medical cannabis. Education on medical cannabis is largely absent in healthcare training programs in Canada, and clinical practice guidelines are important to help inform evidence-based use of cannabis. In March 2021, NICE updated their guideline that made a strong recommendation against use of cannabis to manage chronic pain based on ineffectiveness. Also in March 2021, the International Association for the Study of Pain released a position statement that also strongly recommended against use of cannabis for chronic pain based on low-quality evidence. In September 2021, a guideline was published in the British Medical Journal that found moderate-to-high certainty evidence supporting a weak recommendation in favour of medical cannabis for chronic pain. This presentation will reconcile these contrasting recommendations. Specifically, with a focus on exploring the impact of risk of bias on effect estimates, optimizing presentation of treatment effects to convey benefits and harms in a manner that is most helpful to patients and clinicians, and exploring the critical role of patients’ values and preferences in interpreting the importance of treatment effects and the trade-off between benefits and harms.

**Speaker 2**: Hance Clarke, MD, PhD, University of Toronto, Anesthesiology and Pain Medicine, Toronto, Ontario, Canada, hance.clarke@utoronto.ca @Drhaclarke

**Speaker 2 Abstract Title**: Using real world evidence and basic science to inform the impact of medical cannabis on chronic pain

**Speaker 2 Abstract**: Despite cannabis sales of over 2.6 billion in Canada in 2020, most patients (i.e., 2.7 million of 3.1 million that report using cannabis for a health-related condition) continue to self-medicate with cannabis products intended for recreational sales channels. Only recently has Health Canada enabled Canadian scientists to begin researching the products being consumed by patients in randomized controlled trials. This presentation will update a national real-world evidence project underway and provide early data from the human and basic science studies related to osteoarthritis being completed at the Centre for Cannabinoid Therapeutics (UHN). Pre-clinical study results in our mouse model of osteoarthritis will be presented. THC signals through numerous cannabinoid receptors, including CB1/2 are expressed on joint cells. Our preliminary data suggest that oral delivery methods are superior to intra-articular delivery methods in the mouse model for reducing pain and reducing joint degeneration. Data will also be presented from a cohort of 56 human subjects that were using cannabis to treat chronic pain within the Transitional Pain at the Toronto General Hospital. 70% of the products used by this cohort were considered THC dominant (daily dose:1.7±1.3 grams of product). The majority of patients (96%) reported effective pain management and 76% self-reported a decrease in other analgesic medication usage. Between 83-93% of patients reported symptomatic improvements in nausea, appetite, sleep, anxiety, and depression with medical cannabis use. Compared to males, female patients had numerically higher blood concentrations of CBD, CBDA, Δ^9^-THC, 11-OH-THC but lower concentrations of Δ ^9^-THCA and THC-COOH.

**Speaker 3**: John Brown, Diploma in Police Science, Chronic Pain Centre of Excellence for Canadian Veterans, Dorchester, Ontario, Canada, john.c.brown@me.com

**Speaker 3 Abstract Title**: The role of patient partners in the development of guidance regarding medical cannabis for chronic pain

**Speaker 3 Abstract**: As a Canadian Veteran with lived experience with chronic pain, I have context that is largely unavailable to most researchers and clinicians. This includes my engagement in multiple treatments, including opioids and medical cannabis. I recently participated in the development of a clinical practice guideline on medical cannabis for chronic pain, in which I was a full panel member and co-author. This involved some training to optimize my participation in the process, selecting outcomes of primary interest for evidence syntheses, ranking the relative importance of harms, and helping to establish if effect estimates were imprecise (i.e., did they include both effects that were important and unimportant). The oversight committee engaged 3 patient partners to support the guideline, that were selected in part to represent a variety of experiences. Myself for my positive experience with using medical cannabis, another who had not used cannabis, and another who had tried cannabis but discontinued due to lack of benefit. Each of these perspectives were considered by the panel, as well as a formal review of studies exploring values and preferences of people living with chronic pain towards medical cannabis, when making their recommendation. I will describe my experience with this process to highlight the feasibility and importance of including patient partners with lived experience in the development of clinical practice guidelines that will affect their care.

**Learning Objective 1**: Criticize contrasting guidance recommendations regarding medical cannabis for chronic pain.

**Learning Objective 2**: Demonstrate the importance of generating scientific data that reflects practical use of cannabis for chronic pain.

**Learning Objective 3**: Discuss the critical rolethat patient partners have in the development of guidance for medical cannabis and chronic pain.

**“I feel more confident now and more empowered to give myself more love”: Developing self-management skills and empowering chronic pelvic pain patients**

Rachael Bosma^a^, Tania Di Renna^a^, Wendy Carter^a^ and Emeralda Burke^a^

^a^Women’s College Hospital, Toronto Academic Pain Medicine Institute (TAPMI), Toronto, Ontario, Canada

**Symposium Chair**: Rachael Bosma, PhD, Women’s College Hospital, Toronto Academic Pain Medicine Institute (TAPMI), Toronto, Ontario, Canada, Rachael.Bosma@wchospital.ca

**Symposium Abstract:** Chronic pelvic pain (CPP) is pelvic pain that persists longer than 6 months and is a common, debilitating condition affecting women. CPP occurs in 20% of women of reproductive age and has been recognized as a globally neglected reproductive health morbidity. It accounts for substantial personal suffering and has a direct impact on patient’s marital, social, and professional life. Given the physical, psychological, and social components that contribute to CPP, therapeutic strategies should target both gynecological/biological and psychosocial factors. More specifically, biopsychosocial therapeutic approaches that are based in the educational framework of disease self-management equip patients with the knowledge, resources, and tools to self-manage CPP. Considering that there are very few CPP management programs in Canada and the lengthy wait times for patients, it is important to equip patients with these self-management tools.

This symposium will discuss (1) CPP and CPP management from a biopsychosocial perspective and (2) describe an online *Pelvic Empowered Management Program* (developed by TAPMI) consisting of 6 self-directed modules designed to enhance the self-management of CPP patients who are currently on the waitlist for care at an interdisciplinary chronic pain clinic. We will also consider the experiences, impacts, and outcomes of patients who have completed the *Pelvic Empowered Management Program*. Using audio/video excerpts as well as poignant quotes from our Pelvic Empowered feasibility study, patient stories will be woven in this discussion and described in detail by a patient with lived experience of CPP.

**Speaker 1**: Tania Di Renna, BSc, MD, FRCPC, Women’s College Hospital, Toronto Academic Pain Medicine Institute (TAPMI), Toronto, Ontario, Canada, tania.direnna@wchospital.ca

**Speaker 1 Abstract Title**: The effectiveness of a biopsychosocial self-management approach to chronic pelvic pain

**Speaker 1 Abstract**: Factors contributing to CPP include gynecological, musculoskeletal, visceral, vaginal/vulvar, psychosocial, and central nervous system sensitization. However, up to half of the affected women have no obvious pathology. In addition to their pelvic pain symptoms, women with CPP are at an increased risk for depression, are more likely to have a history of physical and sexual abuse, and subsequently have a higher incidence of posttraumatic stress disorder. These patients often develop negative coping strategies. Management of this patient population is challenging and there are few established treatments. It is not uncommon for patients to undergo multiple consultations and try different pharmacological and surgical therapies. Although CPP may not be curable, it can be managed so patients attain normal or near-normal levels of functions. Providing patients with self-management educational resources is increasingly recognized as a critical strategy for improving self-efficacy and functional outcomes. Self-management reflects the ability to confidently function despite symptoms, including managing medication(s), adapting to physical limitations, and coping with the psychological and emotional challenges associated with one’s disease. Evidence shows self-management support through education can often help patients manage their symptoms more effectively.

This talk will discuss CPP and the importance of educational self-management resources. Throughout the presentation we will address the following questions: 1) Are we providing adequate information and resources to CPP patients? 2) Are we enhancing CPP patients’ ability to self-manage? Through this presentation we will encourage audience participation to facilitate an interdisciplinary discussion on CPP and self-management strategies from a biopsychosocial perspective.

**Speaker 2**: Wendy Carter, C.Psych, Women’s College Hospital, Toronto Academic Pain Medicine Institute (TAPMI), Toronto, Ontario, Canada, wendy.carter@wchospital.ca

**Speaker 2 Abstract Title**: An interdisciplinary and holistic approach to chronic pelvic pain management

**Speaker 2 Abstract**: Treatment for chronic pelvic pain has evolved in the last years to include more modalities as well as more disciplines. As we come to better understand the complexities of chronic pain, especially in the context of pelvic pain, we are in a better position to effectively treat and manage this common condition. There are certain predictive factors involved in chronic pelvic pain such as a history of trauma and life circumstances. Experiencing pelvic pain often comes hand in hand with feelings of isolation and stigma. An interdisciplinary team can provide a holistic approach to care and address some of these layers. An interdisciplinary team is vital in providing the necessary biopsychosocial approach to guide women in developing the self-management skills and tools to live a fulfilled life, despite their pain.

Comprehensive treatment can include one on one treatment as well as group interventions. Topics covered consist of: 1) assessing and addressing any pelvic floor muscle tension and/or weakness by incorporating release techniques and specific exercises, 2) providing education and mindful types of movement in order to increase physical awareness 3) making recommendations on nutrition, and promoting an anti-inflammatory diet, 4) managing emotions and strengthening resilience while cultivating self-compassion, 5) addressing sex and intimacy as well as fertility.

In this session, we will speak to the pelvic pain group for chronic pelvic pain through Toronto Academic Pain Medicine Institute (TAPMI) at Women’s College Hospital.

**Speaker 3**: Rose Robbins, PhD., C. Psych., Psychologist, The Ottawa Hospital Pain Clinic, Ottawa, Ontario, Canada, rorobbins@toh.ca

**Speaker 3 Abstract Title**: A Stepped-care approach to interprofessional pelvic pain management: The Ottawa Hospital Pain Clinic Perspective

**Speaker 3 Abstract**: Access to specialized interprofessional care for chronic pelvic pain remains limited in Canada. Currently many women who live with pelvic pain are treated in general pain clinics either because they have a primary diagnosis of pelvic pain or experience pelvic pain as a comorbidity to another painful condition. Although women with pelvic pain do often benefit from general pain self-management, they have unique needs that are not always addressed effectively in such programs. Their needs are also heterogeneous and require various levels of care.

The Ottawa Hospital Pain Clinic uses a stepped-care approach that has allowed increased access to interprofessional care while reducing wait-times and tailoring treatment to the unique needs of patients. A specific pelvic pain stream was recently created that leverages the existing resources of the clinic to better serve women with pelvic pain. After participating in an intake assessment with an interprofessional team member, patients are given access to several treatment options within two months of their physician referral to the team. These services range from one-time educational workshops to trauma-informed individual therapy. An 8-week interprofessional group treatment facilitated by psychology, physiotherapy, and occupational therapy is also available. In this presentation, we will provide an overview of the pelvic pain stream at The Ottawa Hospital Pain Clinic, including the interprofessional pelvic pain group program and preliminary data on the program’s success in meeting the unique needs of pelvic pain patients.

**Speaker 4**: Emeralda Burke, BSc, Women’s College Hospital, Toronto Academic Pain Medicine Institute (TAPMI), Toronto, Ontario, Canada, emeralda.burke@wchospital.ca

**Speaker 4 Abstract Title**: Empowered Management: The power of lived experience in chronic pelvic pain management and research

**Speaker 4 Abstract**: Living with CPP results in isolation, loneliness and embarrassment due to the challenges associated with this disease, including inability to sit for long periods, difficulty voiding and sexual dysfunction. This leads to mental health struggles and breakdown of relationships as the stigma around talking about such intimate things results in people trying to cope by themselves. However, CPP is present in 10-20% of reproductive-aged women, therefore there needs to be more awareness, education and open discussion about the real-life impact of CPP so that those suffering can understand they are not alone, gain support from their peers and learn to manage their pain. While a general education program is a great foundation, TAPMI saw the potential in creating a targeted educational tool which enables patients to become empowered, feel less alone and understand ways to manage this relentless pain. Our pelvic pain physicians, pelvic physiotherapist, psychologist and several patients living with CPP collaborated to build six self-directed online modules that consisted of educational resources, videos and exercises, called the *Pelvic Empowered Program*.

In this talk, we will provide an overview of the program, the modules, topics covered, and interactive exercises provided to patients. Findings from a study examining the feasibility of this program will be presented and patient experiences with the modules will be described. We want attendees to leave this presentation with an understanding of the impact of the program and the empowerment patients felt in managing their pain from a biopsychosocial lens after completing the program.

**Learning Objective 1**: To discuss chronic pelvic pain from a biopsychosocial perspective and its impact on patient health and quality of life.

**Learning Objective 2**: To discuss interdisciplinary and self-management approaches to chronic pelvic pain management.

**Learning Objective 3**: To describe the experiences, impacts, and outcomes of patients who have completed the Pelvic Empowered Management Program.

**Shift the narrative: Strengths-based and culturally safe considerations for better pain care and policy related to Indigenous Peoples**

Margot Latimer^a^, John Sylliboy^b^, Courtney Pennell^c^ and Katie Gloade^d^

^a^Faculty of Health, Dalhousie University, Halifax, Nova Scotia, Canada; ^b^Education, McGill University, Halifax, Nova Scotia, Canada; ^c^IWK Health, Nursing, Halifax, Nova Scotia, Canada; ^d^Health, Dalhousie University, Halifax, Nova Scotia, Canada

**Symposium Chair**: Margot Latimer, PhD, Nursing, Dalhousie University, Faculty of Health, Halifax, Nova Scotia, Canada, mlatimer@dal.ca @MargotALatimer

**Symposium Abstract:** Indigenous Peoples have knowledge, beliefs, and cultural traditions that keep them healthy. Settler colonial practices cemented by government policies have disrupted Indigenous Peoples’ ability to maintain health and this has left an undeniable, intergenerational, and tragic impact on Indigenous Peoples’ health and well-being. Ongoing oppressive policies and a lack of willingness to uphold treaty and inherent rights have meant that Indigenous peoples have not received equitable, community-informed, and culturally safe care. The Health Council of Canadas landmark document “Empathy Dignity and Respect: Creating Cultural Safety for Indigenous People in Urban Health Care” (2012) clearly identifies significant gaps in healthcare experiences that need to be remedied. There are many real scenarios involving Indigenous People presenting with pain yet are not being treated, or with conditions such as cancer that are ending with tragic results. Research indicates Indigenous peoples feel fearful, disrespected, dismissed, racialized and are reluctant to seek care in the healthcare system. This is especially problematic considering the high rates of multiple, co-existing chronic pain conditions experienced by Indigenous peoples in Canada. Health clinicians perceive Indigenous Peoples health from a deficit based-rather than a strength-based perspective. Shifting the narrative and focusing on strengths will shift our awareness, understanding, and inform our actions to advocate for the creation of safer health spaces for Indigenous Peoples. The symposia bring together the knowledge, experience, and wisdom of three Mi’kmaq people who have worked with Indigenous Peoples in the areas of 2SLGBTQQIA+, education, mental health and trauma and cultural safety in clinical care.

**Speaker 1**: John Sylliboy, M.A, McGill University, Education, Halifax, Nova Scotia, Canada, johnrsylliboy@gmail.com @SylliboyJohnR

**Speaker 1 Abstract Title**: A bundle of knowledge for 2S healthcare

**Speaker 1 Abstract**: Inequities in healthcare also affect the Two-Spirit community. According to a recent survey, Two-Spirit people face an intersection between racism and homophobia, misogyny and transphobia (Wabanaki Two-Spirit Alliance, Sylliboy, 2021). The currently available research demonstrating the intergenerational effects of Indian Residential Schools (IRS) provides support for the enduring negative consequences of these experiences and the role of historical trauma in contributing to present day disparities in well-being. The intersecting and compounded minoritized identities of Two-Spirit people means that they face barriers in healthcare. The varying dimensions of minority stressors faced by Two-Spirit people leads to higher rates of anxiety, depression, PTSD, suicide [ideation], and violent victimization than their straight and/or cisgender peers. There is also evidence this group suffers from more pain conditions due to feeling unsafe and not seeking care in the system. Additionally, the mental health and wellbeing of Two-Spirit people is generally underreported in Canada. Researchers report that significant portions of health care professionals can hold biased and inaccurate beliefs about the causes of health and social inequities and this directly impacts healthcare interactions in the area of pain assessment and treatment.The Wabanaki Two-Spirit Alliance (W2SA) is developing community-led responses to address urgent concerns in this area and are also responding to the MMIWG2S+ Calls for Justice. This presentation will share key, evidence-based promising practices that will enhance the ways health providers can support ways to create safe spaces for the Two-Spirit community in the health care system.

**Speaker 2**: Courtney Pennell, BScN, IWK Health, Nursing, Halifax, Nova Scotia, Canada, courtney.pennell@iwk.nshealth.ca @courtneyPennel3

**Speaker 2 Abstract Title**: Mobilizing indigenous knowledge about pain to create safer healthcare settings for Indigenous People

**Speaker 2 Abstract**: The landmark documents such as the UNDRIP (2007) and TRC Calls to Action (2015), the Canadian Pain Task Force Report (2021) and National Inquiry into Missing and Murdered Indigenous Women and Girls and 2SLGBTQQIA+ reports have identified important information related to culturally safe care in health-care settings. The UNDRIP^3^, first introduced in 2007, is the most comprehensive international instrument outlining the inherent rights of Indigenous peoples to access traditional medicines, maintain their health practices and to be actively involved in developing and determining health outcomes, and designing health programming. There is an urgency to develop knowledge sharing and learning opportunities for health systems, organizations and clinicians to apply this knowledge into practice. Indigenous peoples are in the best position to guide health providers and administrators regarding the path to wellness for Indigenous people and communities. Essential elements of equity-oriented health care for Indigenous Peoples involve partnerships with Indigenous communities, action at all three levels, attention to local and global histories, and attention to potential harmful impacts of different strategies. This presentation will be led by an Indigenous Health Consultant and Registered Nurse who will engage learners to be aware of the ways to create culturally safe, equitable healthcare settings considering system, organization and clinician’s role in terms of pain care. A case study will be presented to provide learners an opportunity to put themselves in a real clinical situation and how these elements fit together.

**Speaker 3**: Katie Gloade, M.Ed, CCC, PhD(c), Dalhousie University, Health, Halifax, Nova Scotia, Canada, katie.gloade@dal.ca @katiegloade

**Speaker 3 Abstract Title**: From curriculum to care: Creating pathways for change

**Speaker 3 Abstract**: As we look to First Nation communities for guidance on how to best support them, we are consistently reminded that the way forward is through collaboration and in keeping with Indigenous Ways of Knowing. The Truth and Reconciliation Commission of Canada: Calls to Action (2015) #22 states that “we call upon those who can effect change within the Canadian healthcare system to recognize the value of Aboriginal healing practices” while call #24 “requires all students” in medical and nursing schools “to take a course dealing with Aboriginal health issues, including the history and legacy of residential schools, the United Nations Declaration on the Rights of Indigenous Peoples, Treaties and Aboriginal rights, and Indigenous teachers and practice” None of these actions, of course, can be done without recognition of the intergenerational impacts of colonization and trauma-informed, culturally safe approaches. Novel work has emerged from a collaboration between First Nations communities and healthcare providers in Mi’kma’ki to demonstrate that First Nations children’s pain is often undiagnosed, untreated, or not referred to specialists. Pain is also experienced on a holistic level and incorporates the physical, mental, emotional, and spiritual dimensions, something that is often not considered in Westernized medicine. Our collective has created an Indigenous-led innovative multi-module cultural safety curriculum relevant for pre-licensure clinicians to learn about landmark documents and ways to apply the knowledge to pain care. This presentation will engage learners to know how to work towards reconciliation in a trauma-informed educational approach so as not to perpetuate harm.

**Learning Objective 1**: Recognize how colonizing historical events such as Indian Residential School and Indian Day School impact Indigenous Peoples current health and healthcare pain experiences.

**Learning Objective 2**: Recognize why and how to meaningfully engage Indigenous Peoples in trauma-informed and culturally safe health care.

**Learning Objective 3**: Identify ways that clinicians and organizations can use evidence and landmark reports such as the Truth and Reconciliation Commission (TRC) Calls to Action and the United Nations Declaration on the Rights of Indigenous Peoples (UNDRIP), Missing and Murdered and Indigenous Women and Girls and 2SLGBTQQIA+ to create stronger policy that create trauma-informed health spaces that recognize Indigenous Peoples knowledge.

**The role of the gut microbiome in chronic pain - mechanisms and clinical implications**

Arkady Khoutorsky^a^, Amir Minerbi^b^, Weihua Cai^c^ and Yoram Shir^d^

^a^McGill University, Montreal, Quebec, Canada; ^b^Institute for Pain Medicine, Rambam Health Campus, Haifa, Israel; ^c^Anesthesia, McGill University, Montreal, Quebec, Canada; ^d^The Alan Edwards Pain Management Unit, McGill University Health Centre, McGill University, Montreal, Quebec, Canada

**Symposium Chair**: Arkady Khoutorsky, PhD, McGill University, Montreal, Quebec, Canada, arkady.khoutorsky@mcgill.ca

**Symposium Abstract:** Changes in the gut microbiome have been recently observed in several chronic pain conditions, including visceral pain and chronic widespread pain. Accumulating evidence in human and animal studies suggests that these changes might contribute to the disease pathophysiology and enhance pain hypersensitivity, not only in these pain conditions but also in neuropathic pain. The underlying molecular mechanisms, however, are poorly understood. In this session, we will overview the field of the gut microbiome in chronic pain and discuss ongoing studies on the gut microbiome in different chronic pain conditions in humans. Specifically, we will present results supporting the causal role of the gut microbiome in fibromyalgia, providing evidence for: 1) alterations in gut microbiome composition and function in patients with fibromyalgia, and 2) the causal role of fibromyalgia-associated gut bacteria in causing pain hypersensitivity in animal models. Finally, we will discuss potential underlying mechanisms that could explain the role of the microbiome in pain, highlighting potential clinical implications of this new field, as well as its promising future directions.

**Speaker 1**: Amir Minerbi, MD PhD, Institute for Pain Medicine, Rambam Health Campus, Haifa, Israel, minerbi@tehcnion.ac.il,

**Speaker 1 Abstract Title**: Gut microbiome alterations in fibromyalgia are associated with changes in serum bile acid profile and symptom severity

**Speaker 1 Abstract**: In recent years, there has been growing appreciation of the critical role the gut microbiome plays in health and disease. This rich ecosystem of micro-organisms living in and on our body is not only modelled by a variety of medical conditions, but also plays a role in pathologies pertaining to a wide range of medical fields. Could the gut microbiome play a role in chronic pain as well? To test this hypothesis, we explored the composition and function of the gut microbiome in a cohort of 77 women with fibromyalgia and 79 healthy controls. While the overall composition of the gut microbiome of patients was similar to that of controls, significant alterations were observed in the relative abundance of several specific bacterial species. Serum metabolomic analysis revealed substantial alterations in the concentration of gut-microbiome-derived metabolites, including short-chain fatty acids (SCFA) and bile acids. These differentially abundant serum metabolites are known to be metabolized by the differentially abundant bacterial species identified in fibromyalgia patients, indicating an altered gut-microbiome function in addition to the observed alteration in its composition. Moreover, gut microbiome composition and serum bile acid concentrations were highly correlated with symptom severity, including pain intensity and fatigue. The changes observed in the composition of the gut microbiota, and the expression of circulating secondary bile acids seem congruent with the phenotype of increased nociception. These results demonstrate a unique biological signature of the gut microbiome and circulating bacterial metabolic end-products in patients with fibromyalgia.

**Speaker 2**: Weihua Cai, PhD, McGill University, Anesthesia, Montreal, Quebec, Canada, weihua.cai@mail.mcgill.ca

**Speaker 2 Abstract Title**: Fecal microbiome transplantation from fibromyalgia patients induces disease-like symptoms in mice

**Speaker 2 Abstract**: The gut microbiota consists of a diverse and dynamic community of microorganisms that inhabit the gastrointestinal tract and plays a role in host health and disease. Dysregulation of the gut microbial community has been linked to intestinal, metabolic, neurological, and psychiatric disorders. It has been recently shown that the composition of the gut microbiota is altered in individuals with fibromyalgia. Fibromyalgia is characterized by chronic widespread pain, coupled with fatigue, sleep disturbances and cognitive dysfunction. To study the causal role of the altered gut microbiota in the development of this syndrome, we performed fecal microbiota transplantation from patients with fibromyalgia and healthy controls to germ-free mice and measured mechanical and thermal sensitivity, and spontaneous pain. We also assessed general activity, memory functions, anxiety, and depression. Moreover, we performed comprehensive molecular analyses, including metabolomics, sequencing of different tissues and feces (16S), and assessment of peripheral blood mononuclear cell (PBMC) composition and neuroinflammation in the central nervous system. During this talk, I will present the results from these studies, showing that the gut microbiome plays a causal role in the fibromyalgia pathophysiology and discuss potential underlying mechanisms.

**Speaker 3**: Yoram Shir, MD, McGill University, The Alan Edwards Pain Management Unit, McGill University Health Centre, Montreal, Quebec, Canada, yoram.shir.med@ssss.gouv.qc.ca

**Speaker 3 Abstract Title**: Hopes for the future: can the gut microbiome be harnessed to improve chronic pain therapy?

**Speaker 3 Abstract**: At present, chronic pain is predominantly a cureless disease. This unfortunate reality stems from multitude reasons, including, but not limited to, our lack of understanding of basic pain mechanisms, lack of appropriate diagnostic tools, ignoring the role of the environment, inability to find meaningful association with genetic factors, lack of specific therapeutic modalities and focusing on pain palliation rather than prevention. Thus we, the frustrated clinicians, are left with no choice but to treat patients with the same non-specific, crude and many times ineffective therapeutic modalities. This dire state of affairs calls for exploring and developing new different, non-traditional tools to prevent and treat chronic pain. Gut microbiota play a critical role in diverse biological processes, including regulating neurologic signaling and neurotransmitters, and modifying the response to drugs. Its dysbiosis could be associated with neurologic and psychiatric disorders and with chronic pain conditions. We, therefore, believe that gut microbiome could be directly or indirectly involved in the development and maintenance of chronic pain. In my talk, I will touch on few possible future research directions to further establish this assumption, including: 1) exploring the causality of dysbiosis in chronic pain; 2) screening patients with variety of chronic pain conditions to establish specific microbiome/pain trajectories; 3) exploring whether gut bacteria diversities and/or metabolomic changes could serve as diagnostic markers for specific pain conditions; 4) manipulating the composition of the gut microbiome as a potential therapeutic tool; 5) pre-emptively changing the microbiome to prevent the development of chronic pain.

**Learning Objective 1**: Upon completion of this session, attendees will be able to describe the altered microbiome composition observed in humans with fibromyalgia, accompanied by changes in circulating bacterial metabolic end-products.

**Learning Objective 2**: Upon completion of this session, attendees will be able to describe the role of the gut microbiome from fibromyalgia patients in causing pain hypersensitivity in rodents.

**Learning Objective 3**: Upon completion of this session, attendees will be able to describe the limitations of the currently available diagnostic and therapeutic tools to treat chronic pain, and the hope that the gut microbiome could be harnessed to improve our ability to prevent, diagn.

**Innovations in primary dysmenorrhea across the lifespan: Perspectives on conceptualization and treatment**

Michelle M. Gagnon^a^, Kayla Wall^a^ and Nicole M. Alberts^b^

^a^Department of Psychology and Health Studies, University of Saskatchewan, Saskatoon, Saskatchewan, Canada; ^b^Department of Psychology, Concordia University, Montreal, Quebec, Canada

**Symposium Chair**: Michelle M. Gagnon, Ph.D., University of Saskatchewan, Department of Psychology and Health Studies, Saskatoon, Saskatchewan, Canada, michelle.gagnon@usask.ca @MicheGagnon

**Symposium Abstract:** Dysmenorrhea, or menstrual pain, is the most common cause of pain in females of reproductive age. Researchers have reported prevalence rates of primary dysmenorrhea in girls and women to range from approximately 40% to over 90%. Moreover, one in four adolescents who menstruate and one in five adults who menstruate rate their pain as impairing. Despite a large proportion of individuals who menstruate experiencing clinically significant menstrual pain on a regular basis, dismissal of menstrual pain remains a major societal concern. The perception that menstrual pain is a “normal” part of life remains pervasive and many individuals who do seek help for their pain are too often dismissed. Interventions targeting pain with menstruation are limited, primarily pharmacological, and often inaccessible by individuals who menstruate. Psychological interventions that are effective in managing other pain conditions have remained virtually ignored in the context of menstrual pain. Individuals who menstruate deserve access to high-quality care that decreases the impact of pain on their daily lives and functioning. We will shed light on experiences and treatment needs for individuals with primary dysmenorrhea, with focus on improving conceptualization, understanding overlooked avenues for treatment, and presenting novel digital health-based interventions for youth with primary dysmenorrhea.

**Speaker 1**: Michelle M. Gagnon, Ph.D., University of Saskatchewan, Department of Psychology and Health Studies, Saskatoon, Saskatchewan, Canada, michelle.gagnon@usask.ca @MicheGagnon

**Speaker 1 Abstract Title**: Conceptualizing primary dysmenorrhea across the lifespan from a biopsychosocial perspective: What we know and what is still needed

**Speaker 1 Abstract**: Many individuals who menstruate suffer through menstrual pain because they believe that pain is a typical part of the female menstrual cycle that they must endure. The consequences of dysmenorrhea are not trivial, and can include regular incapacitating symptoms, increases in mental health symptoms, lower quality of life, and interference with work and school functioning. Nevertheless, primary dysmenorrhea has received little attention compared to other pain and gynecological conditions. Biopsychosocial models of pain emphasize the importance of psychological and social contributors to pain; however our recent work has identified a significant lack of consideration of such factors in the conceptualization and treatment of primary dysmenorrhea. This presentation will review findings from our ongoing program of research and will focus on the psychological and social influences in primary dysmenorrhea. Additionally, lifespan influences on dysmenorrhea will be discussed to provide an overview of what we have learned about factors that influence primary dysmenorrhea across the reproductive years. Integrating findings from our research and research from other groups, recommendations for future research and practice will be provided.

**Speaker 2**: Kayla Wall, M.Sc., University of Saskatchewan, Department of Psychology and Health Studies, Saskatoon, Saskatchewan, Canada, kayla.wall@usask.ca

**Speaker 2 Abstract Title**: Treatment preferences and experiences of adolescents and young adults with primary dysmenorrhea

**Speaker 2 Abstract**: Despite the prevalence of primary dysmenorrhea during adolescence, little is known about adolescents’ treatment needs and experiences for this condition. Current consensus guidelines for the treatment of primary dysmenorrhea (e.g., Society of Obstetricians and Gynecologists of Canada) recommend pharmacological interventions (e.g., NSAIDs or oral contraceptives) as the first line treatment. Yet, pharmacological interventions are not always accessible to adolescents and up to 25% of individuals do not experience symptom relief from pharmacological treatments. Further, psychological interventions are not included in treatment recommendations, which is misaligned with current recommendations for chronic and persistent pain conditions. There is a need to better understand adolescents’ experiences with pharmacological interventions as well as their interest in exploring alternative treatment options, such as psychological options, to improve dysmenorrhea care. We recruited a nation-wide sample of young adults between the ages of 18 and 25 to examine young adults’ retrospective account of their experience receiving treatment for their menstrual pain throughout their adolescence. This presentation will review our findings regarding (1) experiences with treatment for dysmenorrhea in adolescents, including barriers and facilitators to seeking treatment, (2) treatment preferences and recommendations for improvements to existing treatment options, and (3) perceptions and openness to engaging in psychological intervention to manage their menstrual pain during adolescence. The clinical implications and potential future directions for these findings will also be discussed.

**Speaker 3**: Nicole M. Alberts, Ph.D., Concordia University, Department of Psychology, Montreal, Quebec, Canada, nicole.alberts@concordia.ca @NAlbertsPhD

**Speaker 3 Abstract Title**: Menstrual pain management across the lifespan: Leveraging digital health and user-centered design to improve access and outcomes

**Speaker 3 Abstract**: Menstrual pain is prevalent among adolescents and adults – with up to 90% of adolescents who menstruate experiencing menstrual pain and a quarter of these individuals reporting their pain to be severe or very severe. Psychological therapies are effective at reducing pain among adolescents and adults with chronic or persistent pain – yet many barriers to accessing this care exist. Digital health interventions have the potential to improve access to psychological treatments for menstrual pain and to overcome barriers to care, including geographical distance, stigma, cost, and lack of trained providers. In this presentation, we will first present data from a scoping review of the content and quality of smartphone apps targeting menstrual pain and symptoms across the lifespan. Overall, results of this review showed a lack of self-management content within the identified apps and low overall app quality. Additionally, only one app was designed specifically for adolescents. Next, we will describe the user-centered design and development of a new app-based self-management intervention targeting menstrual pain among adolescents. Specifically, a three-phase approach will be discussed including: 1) identification of adolescents’ treatment needs and preferences, 2) usability testing and app refinement, and 3) feasibility testing to examine adolescents’ satisfaction with the app and preliminary treatment outcomes. Clinical implications and future research directions around the use of digital health interventions to target menstrual pain will be discussed. Recommendations and potential challenges with respect to generating appropriate and engaging intervention content for both adolescents and adults with menstrual pain will also be outlined.

**Learning Objective 1**: At the end of this session, attendees will be able to describe the psychological and social factors that influence primary dysmenorrhea experiences across the lifespan.

**Learning Objective 2**: At the end of the session, attendees will be able to recognize the treatment needs and interests of adolescents and young adults with primary dysmenorrhea.

**Learning Objective 3**: At the end of the session, attendees will be able to discuss how psychological and digital interventions can be leveraged to treat primary dysmenorrhea.

**Central mechanisms of pediatric chronic pain: insights from novel neuroimaging studies**

Melanie Noel^a^, Laura Simons^b^, Marina Lopez Sola^c^ and Massieh Moayedi^d^

^a^University of Calgary, Calgary, Alberta, Canada; ^b^Stanford University, Palo Alto, California, USA; ^c^University of Barcelona, Barcelona, Spain; ^d^University of Toronto, Toronto, Ontario, Canada

**Symposium Chair**: Melanie Noel, PhD, RPsych, University of Calgary, Calgary, Alberta, Canada, melanie.noel@ucalgary.ca @MelanieNoel

**Symposium Abstract:** Chronic pain is a common childhood problem and can profoundly impact childrens physical, psychological and social functioning. It affects one in five children, and often emerges in adolescence and persists into adulthood: 2/3 of youth with chronic pain become adults with chronic pain. Current treatments for chronic pain are suboptimal and have been tied to the opioid crisis. The nature and severity of pediatric pain varies with age and sex. Recent research has substantially advanced our understanding of the pathogenesis of these conditions. However, there are limited data to inform mechanism-based understanding and management of pain in adolescents. This workshop will present new research findings shedding mechanistic insight on pediatric pain and discuss novel behavioural, psychological, and neural risk factors associated with treatment responsiveness. Each talk brings forth novel approaches to investigate mechanisms of chronic pain. The chair, Dr. Melanie Noel, will formally lead a group discussion, and synthesize the evidence presented across the talks, and bring her expertise to inform the mechanisms and framworks discussed. Together, these talks will highlight the heterogeneity of pediatric chronic pain. and the differing mechanisms contributing to these various phenotypes. They will provide a multimodal approach to understanding and treating pediatric chronic pain.

**Speaker 1**: Laura Simons, MS, PhD, Stanford University, Palo Alto, California, United States, lesimons@stanford.edu, @Laura_Simons

**Speaker 1 Abstract Title**: Pain stickiness: Predicting recovery or persistence in pediatric Complex Regional Pain

**Speaker 1 Abstract**: Only ~50% of adolescents with chronic pain who present for multidisciplinary pain treatment recover, as measured by clinical endpoints of pain severity and functional disability. Discovery of robust markers of the recovery vs. persistence of pain and disability is essential to develop more resourceful and patient-specific treatment strategies and to conceive novel approaches that benefit patients who are refractory. Given that chronic pain is a biopsychosocial process, the discovery and validation of a prognostic and robust signature for pain recovery vs. persistence requires measurements across multiple dimensions. This presentation will include introduction of brief screening tools for youth with chronic pain and their parents to rapidly assess risk factors and enhance targeted treatment allocation. Moreover, it will include neurobiological risk factors of brain structure and functional patterns in relation to responsivity to treatment in youth with complex regional pain syndrome. Overall, the presentation will take into account psychosocial and neurobiological factors associated with treatment responsiveness on pain in youth.

**Speaker 2**: Marina Lopez Sola, MS, PhD, University of Barcelona, Barcelona, Spain, mlopezsola@ub.edu, @mlopezsola82

**Speaker 2 Abstract Title**: Neurophysiological and psychological mechanisms of fibromyalgia across the lifespan: understanding adult and juvenile forms of the disease

**Speaker 2 Abstract**: Fibromyalgia (FM) is a debilitating, chronic pain condition affecting primarily females. Although we are used to thinking about fibromyalgia as a disease affecting people in their 40s and 50s, it is also highly prevalent amongst children and adolescents, with 2-6% of schoolers affected. The majority of patients suffering from juvenile fibromyalgia during this critical period for brain development continue to experience persistent pain during adulthood. Due to the lack of physiological laboratory findings, both FM and JFM have been questioned as clinical entities and have been frequently regarded as merely an expression of anxiety or depression. This leads to poor understanding, stigmatization, and inappropriate disease management, underscoring the need for identifying objective pathophysiology early on. In this workshop we will present published evidence from the adult literature on the neurophysiology of adult fibromyalgia emphasizing brain correlates that are highly predictive of the disorder in the domains of pain, non-painful multisensory processing and emotional/cognitive processing. We will also present novel results on the neurophysiological and psychological mechanisms underlying juvenile fibromyalgia and brain correlates underlying different symptom dimensions. We will offer a qualitatively integrated perspective on brain and psychological findings overlap and separability between juvenile and adult forms of the disease.

**Speaker 3**: Massieh Moayedi, PhD, University of Toronto, Toronto, Ontario, Canada, m.moayedi@utoronto.ca @massihmoayedi

**Speaker 3 Abstract Title**: Developmental trajectories of pain-related brain regions in health and in neuropathic pain

**Speaker 3 Abstract**: Multimodal assessment and phenotyping is common in adults with neuropathic pain. In adolescents, however, neuropathic pain is associated with significant pain and pain-related disability, but the sources and causes of pain can differ from those in adults. Neuropathic pain is rarely systematically studied in children and young people, and the causes of the disease can vary from those in adults. Neuropathic pain in adults has been associated with widespread neural changes. However, the brains of children and adolescents undergo significant developmental changes. The impact of chronic pain on such rapidly changing brains remains unknown. Furthermore, there is very limited evidence about normative development of functional brain networks, including those involved in nociception and pain. Dr. Moayedi will discuss new findings from normative structural and functional developmental trajectories of brain regions involved in nociception and pain modulation. Next, he will discuss the feasibility and acceptability of MRI scanning in children with neuropathic pain, and present novel findings of brain functional connectivity abnormalities in children with neuropathic pain. These data provide novel insights into the disease mechanisms of pediatric neuropathic pain, and potential novel therapeutic targets.

**Learning Objective 1**: Develop an understanding of the complexity and heterogeneity of pediatric chronic pain, and the underlying mechanisms.

**Learning Objective 2**: Identify novel methods for phenotyping pediatric pain patients.

**Learning Objective 3**: Learn how a precision medicine approach can improve treatment outcomes.

**An update on neuropathic pain across the lifespan**

Jennifer Stinson^a^, Anuj Bhatia^b^, Vina Mohabir^c^ and Giulia Mesaroli^a^

^a^The Hospital for Sick Children, Toronto, Ontario, Canada; ^b^University of Toronto and Toronto Western Hospital, Toronto, Ontario, Canada; ^c^SickKids Research Institute, The Hospital for Sick Children, Toronto, Ontario, Canada

**Symposium Chair**: Dr Jennifer Stinson, RN PhD, The Hospital for Sick Children, Toronto, Ontario, Canada, jennifer.stinson@sickkids.ca

**Symposium Abstract:** Neuropathic pain has a population prevalence of 4-8%, it often requires complex management, and it is often refractory to conventional therapies. It accounts for approximately 30% of referrals to tertiary care pain clinics. There have been significant advances in our understanding, diagnosis, and treatment of neuropathic pain over the last decade. In 2011, neuropathic pain was redefined by the International Association for the Study of Pain as ‘pain that arises from damage or disease of the somatosensory nervous system’, no longer encompassing pain from nervous system dysfunction (later termed nociplastic pain in 2016). This change in definition has important implications for diagnosis and this will be discussed in the symposium including the framework for screening and diagnosing neuropathic pain as possible, probable, and definite. Several advances in the treatment of neuropathic pain including pharmacological (e.g., long-acting gabapentinoids, cannabinoids), interventional (e.g., perineural steroids and botulinum, spinal cord and peripheral nerve neuromodulation), and rehabilitation (e.g., desensitization, graded motor imagery) will also be discussed. Presenters Dr Bhatia and Ms Mesaroli will provide an update on the current understanding of neuropathic pain, approach to screening, diagnosis, and treatment (pharmacological, interventional, rehabilitation). Dr Bhatia will speak to neuropathic pain in the adult population and Ms Mesaroli in the pediatric population. Ms Mohabir will provide a lived experience lens to neuropathic pain after experiencing neuropathic pain from adolescence to adulthood.Audience members will be engaged throughout the symposium by using interactive technologies such as Poll Anywhere and dedicated time (15 min) for questions for speakers.

**Speaker 1**: Dr Anuj Bhatia, MBBS MD PhD FRCPC (Anesthesia and Pain Medicine), University of Toronto and Toronto Western Hospital, Toronto, Ontario, Canada, anuj.bhatia@uhn.ca

**Speaker 1 Abstract Title**: An update on neuropathic care in adults

**Speaker 1 Abstract**: Neuropathic pain (NP) in adults is often severely debilitating, and its management and sequelae are a significant burden on health care resources. Knowledge gaps exist for health-care providers caring for patients with NP. A graded system has been proposed to determine the level of certainty with which the pain in question is neuropathic (and not nociceptive). The rationale and features of this system will be presented in the symposium along with the criteria for labeling NP as ‘possible’, ‘probable’, or ‘definite’. The characteristic symptoms and signs of NP have led to the development of several validated questionnaires to screen for and assess it in clinical practice and research. Diagnostic characteristics of these instruments will be presented in the symposium. Investigations for evaluating somatosensory pathway function including quantitative sensory testing, nerve conduction studies, skin biopsy and corneal confocal microscopy will be elaborated. Treatment strategies for NP including pharmacological options, physical therapy, and cognitive behavioral interventions will be presented in the symposium. The role of more invasive therapies including perineural interventions and neuromodulation approaches (spinal cord or peripheral nerve stimulation and intrathecal therapies) in treating refractory NP in adults will also be discussed.

**Speaker 2**: Vina Mohabir, BSc(Hons), SickKids Research Institute, The Hospital for Sick Children, Toronto, Ontario, Canada, vina.mohabir@gmail.com @VinaMohabir

**Speaker 2 Abstract Title**: Living with neuropathic pain

**Speaker 2 Abstract**: Ms. Mohabir will provide a lived experience perspective on the content presented by both Dr. Bhatia and Ms. Mesaroli. As a teenager, Ms. Mohabir was injured by a softball. Unfortunately, this led to trigeminal neuralgia and chronic migraine. At the time, it was challenging to find healthcare providers who could understand her neuropathic pain. Eventually, Ms. Mohabir was able to access multidisciplinary pain treatment at the Hospital for Sick Children Chronic Pain Clinic. It was the first time Ms. Mohabir, her parents, and siblings had heard about neuropathic pain. Ms. Mohabir credits the 3P method to chronic pain treatment (psychological intervention, physical/rehabilitative methods, and pharmacological approaches). Using rehabilitative treatment methods, she was able to resume some parts of a normal adolescence. She was able to cope well for many years before a spinal cord injury. Treatment for neuropathic pain in adult care had different challenges and treatment options. She was treated at the Toronto Academic Pain Medicine Institute (TAPMI), where she received pharmacological (e.g., pregabalin, cannabinoids), interventional (e.g., steroids, spinal cord neuromodulation), and rehabilitation (e.g., desensitization, graded motor imagery) treatment. She copes with neuropathic pain every day using these methods – but is able to work, have a social life, travel, hike, and enjoy time with her rescue dog Milo.

**Speaker 3**: Giulia Mesaroli, MScPT, BASc, The Hospital for Sick Children, Toronto, Ontario, Canada, giulia.mesaroli@sickkids.ca @GMesaroli

**Speaker 3 Abstract Title**: Neuropathic pain in children and adolescents

**Speaker 3 Abstract**: Neuropathic pain (NP) in children is particularly problematic as it is associated with significant pain-related disability including social isolation, school absenteeism, physical disability, sleep and mood disorders, and is particularly costly to the health care system. NP in pediatrics is unique from that of adults as the etiology is more diverse and patterns of pain experience, coping strategies, and cognitive development vary significantly throughout childhood. Common causes of adult NP such as diabetic neuropathy and postherpetic neuralgia are much less common in pediatrics. Causes of pediatric NP are highly diverse and include trauma, cancer, infections, genetic and neurological disorders.Ms. Mesaroli will present an overview of neuropathic pain conditions in the pediatric setting informed by a scoping review of the literature. The prevalence, clinical features, age and sex-based differences in pediatric NP conditions will be discussed. She will also highlight the challenges of screening and diagnosis for NP in the pediatric setting: (1) currently available screening tools were developed and validated in adults and (2) the new ICD-11 diagnostic codes do not reflect common causes of pediatric NP. A novel screening tool (Pediatric PainSCAN©) for pediatric NP will be introduced, developed by speakers G.M. and J.S. Rehabilitative treatment approaches for pediatric NP will be reviewed, including pain neuroscience education, graded motor imagery (e.g., laterality, visualization, and mirror therapy), sensory strategies (e.g., desensitization), virtual reality, and graded exercise.

**Learning Objective 1**: To identify the current approach to screening and diagnosing neuropathic pain in children and adults.

**Learning Objective 2**: To describe multi-modal treatment approaches to neuropathic pain in children and adults.

**Learning Objective 3**: To recognize the impact of neuropathic pain on children and adults - and the role of multidisciplinary treatment in coping with neuropathic pain.

**Update on mechanisms of craniofacial pain and their clinical correlates**

Brian Cairns^a^, Barry Sessle^b^ and Carolina Beraldo Meloto^c^

^a^Faculty of Pharmaceutical Sciences, University of British Columbia, Vancouver, British Columbia, Canada; ^b^Faculty of Dentistry, University of Toronto, Toronto, Ontario, Canada;^c^Faculty of Dentistry, McGill University, Montreal, Quebec, Canada

**Symposium Chair**: Barry Sessle, MDS, PhD, DSc (h.c.), University of Toronto, Dentistry, Toronto, Ontario, Canada, barry.sessle@utoronto.ca

**Symposium Abstract:** Acute and chronic pain conditions in the face or mouth are very common, and some are unique to the craniofacial region. However, many of the pain conditions, especially those that are chronic, are difficult to diagnose and manage, and their aetiology and pathogenesis are unresolved. This Symposium will review recent findings bearing on underlying mechanisms and factors influencing craniofacial pain, and outline their clinical implications. The presentation ‘Peripheral Trigeminal Pain Mechanisms’ by Dr BE Cairns (UBC) will explore what is known about peripheral pain mechanisms in craniofacial tissues and how this knowledge is being used to identify novel analgesic targets. The topic ‘Central Trigeminal Pain Mechanisms’ presented by Dr BJ Sessle (UToronto) will provide an overview of the mechanisms within the brain that underlie craniofacial pain, and also note the relevance of these findings to the diagnosis and management of craniofacial pain conditions. The presentation ‘Genetics of Craniofacial Pain’ by Dr. CB Meloto (McGill U) will discuss how genetics can be used to reconstruct the mechanisms underlying craniofacial pain, as well as what other health research fields can teach us about the applicability of genetic findings to clinical care.

**Speaker 1**: Brian Cairns, BSc, BSc(Pharm), PhD, DrMed, ACPR, University of British Columbia, Faculty of Pharmaceutical Sciences, Vancouver, British Columbia, Canada, brian.cairns@ubc.ca

**Speaker 1 Abstract Title**: Peripheral mechanisms of trigeminal pain

**Speaker 1 Abstract**: Pain signals from craniofacial tissues are transmitted to the central nervous system by slowly conducting myelinated and unmyelinated afferent fibers whose cell bodies are located in the trigeminal ganglion. These trigeminal nociceptors are characterized by having non-specialized endings, which respond to high threshold mechanical and thermal stimuli, as well as a variety of noxious chemical stimuli. The endings of these nociceptors contain neurotransmitters (glutamate) and neuropeptides (CGRP, substance P, PACAP etc), which may be released to induce a localized vasodilation and afferent sensitization. In addition, the terminal endings of trigeminal nociceptors have been found to express receptors for neurotransmitters such as glutamate, GABA, serotonin, noradrenaline, and purines as well as for various neuropeptides. Glutamate, noradrenaline, serotonin, and adenosine triphosphate sensitize trigeminal nociceptors through NMDA, a 1 adrenergic, 5HT 1a,b,& 3 and P 2 X receptors, respectively, while GABA, although it appears to depolarize afferent endings, causes desensitization. Recent work has shown that trigeminal ganglion neurons also express these same receptors, and that ganglion neurons as well as their associated satellite glial cells, can release neurotransmitters and neuropeptides. In vivo administration of glutamate into the trigeminal ganglion excites ganglion neurons, and induces a delayed mechanical sensitization of their peripheral endings. In contrast, in vivo administration of GABA has no effect on ganglion neurons, but appears to induce a delayed mechanical desensitization of their peripheral endings. These and other findings suggest that agents which alter peripheral neuroreceptor function modulate nociceptive signals from craniofacial tissues by acting at their terminal endings and also within the trigeminal ganglion.

**Speaker 2**: Barry Sessle, MDS, PhD, DSc (h.c.), University of Toronto, Faculty of Dentistry, Toronto, Ontario, Canada, barry.sessle@utoronto.ca

**Speaker 2 Abstract Title**: Central trigeminal pain mechanisms

**Speaker 2 Abstract**: Studies in rodent models of craniofacial pain have revealed that craniofacial injury or inflammation induces abnormal hyperexcitable or ectopic primary afferent inputs to the central nervous system (CNS) that produce neuroplastic changes expressed as an increased excitability of nociceptive neurons within trigeminal nociceptive CNS circuits. This “central sensitisation” has been documented as immunohistochemical changes as well as electrophysiological alterations in the receptive field and response properties of the nociceptive neurons that contribute to the accompanying nociceptive behaviour in the rodent models; this behaviour reflects allodynia, hyperalgesia, pain spread or spontaneous pain that are typical clinical features of many types of chronic pain. Several mediators, receptors and signalling mechanisms have been identified as crucially involved in the production of the increased excitability of the nociceptive neurons. These include glutamatergic, neurokinin (eg, substance P; CGRP) and purinergic (e.g., ATP) mediators released from the CNS endings of the primary afferents, intracellular signalling processes such as nitric oxide and pERK, as well as mediators such as cytokines released from other cells (e.g., glia). These processes are potential targets for the development of novel analgesic agents or the re-purposing for pain control of agents currently used for other biomedical conditions. Also notable are recent findings in rodent craniofacial pain models that the predisposition to nociceptive behaviour as well as the accompanying trigeminal central sensitisation show considerable variability between genetically different rodent strains. This is consistent with findings from chronic craniofacial pain states in humans that genetic factors may contribute to pain expression or its predisposition.

**Speaker 3**: Carolina Beraldo Meloto, MSc, DDS, PhD, McGill University, Faculty of Dentistry, Montreal, Quebec, Canada, carol.meloto@mcgill.ca

**Speaker 3 Abstract Title**: Genetics of craniofacial pain

**Speaker 3 Abstract**: Chronic craniofacial pain conditions are disabling health problems that are poorly managed largely because our understanding of its pathophysiological mechanisms still is incomplete and available treatments are hence unspecific. In this presentation, I will discuss how genome-wide based approaches can aid in the reconstruction of these mechanisms. Specifically, pathway analysis based on genome-wide association data of four independent case-control studies of chronic temporomandibular disorders, the most frequent form of chronic craniofacial pain condition, has implicated trigeminal nerve morphogenesis and semaphoring-plexin axonal guidance in the pathophysiology of this condition. Genes in these pathways are semaphorins, plexins and neuropilins that have canonical axonal guidance roles, suggesting that genetically driven abnormalities during CNV morphogenesis may contribute to TMD. Subsequent studies using an unprecedented neuroimaging approach begin to show differences in the shape and connectivity of the CNVs of people with or without TMD. Preclinical studies using mice with impaired semaphoring-signalling and consequent morpho-structural abnormalities in the CNV sensory pathways also show that these animals have increased susceptibility to orofacial pain. These findings are an example of how genetics can be used to reveal the pathophysiology of craniofacial pain. I will additionally discuss the current knowledge on the genetics of TMD and other common craniofacial pain conditions and how genetics may aid in the clinical care of chronic pain patients in the near future.

**Learning Objective 1**: Describe recent advances in understanding of the neural processes in craniofacial tissues and the brain that are involved in the development and maintenance of craniofacial pain.

**Learning Objective 2**: Document the role of molecular mechanisms involving glioplasticity as well as genetic factors in the expression of craniofacial pain and its control.

**Learning Objective 3**: Identify the implications of these recent findings for the diagnosis and novel management of craniofacial pain conditions.

**Pain and aging: Developments in basic and clinical sciences**

Jeffrey S. Mogil^a^, Thomas Hadjistavropoulos^b^ and Mary-Ann Fitzcharles^c^

^a^McGill University, Montreal, Quebec, Canada; ^b^Psychology and Centre on Aging and Health, University of Regina, Regina, Saskatchewan, Canada; ^c^Faculty of Medicine, McGill University, Montreal, Quebec, Canada

**Symposium Chair**: Thomas Hadjistavropoulos, Ph.D., FCAHS, University of Regina, Psychology and Centre on Aging and Health, Regina, Saskatchewan, Canada, thomas.hadjistavropoulos@uregina.ca @URHealthPsycLab

**Symposium Abstract:** The primary objective of this symposium is to provide an update on recent developments in pain and aging from the perspectives of basic science, assessment and clinical management. From a basic science standpoint, we will present new data from animal research on the differential impact of pain on lifespan as a function of sex. In regards to assessment, we will present recent development and evaluation of advanced technologies (e.g., computer vision) designed to identify and monitor pain in older adults with limited ability to communicate due to advanced dementia. From a clinical science standpoint, we will review the increasing interest and research on the effects of cannabis on older adults with chronic pain and will present recommendations for clinicians.

**Speaker 1**: Jeffrey S. Mogil, PhD, FCAHS, FRSC, McGill University, Montreal, Quebec, Canada, jeffrey.mogil@mcgill.ca @JeffreyMogil

**Speaker 1 Abstract Title**: Pain, sex, and death

**Speaker 1 Abstract**: A major reason pain is not taken as seriously as it should is that it causes morbidity but not mortality. However, epidemiological studies using large data sets like the UK Biobank have revealed that chronic pain is associated with excess mortality risk. In an attempt to uncover the mechanisms underlying this relationship, we have been studying mice at time points long past the usual time span of preclinical pain studies. We observe that no earlier than 4 months after a spared nerve injury (SNI)—which causes persistent neuropathic pain—male but not female mice exhibit telomere length decreases and cellular senescence in spinal cord microglia. This senescence appears to maintain pain behaviour, since clearing of the senescent cells reverses such behaviour. Male and female mice display very different trajectories of pain over long periods of time, with stable evidence of pain until death in male mice, and a recovery followed by a renewal of pain behaviour in female mice. Although we found no significant effect of SNI on lifespan in either sex, in male but not female mice the average level of pain behaviour over 2–3 years is significantly and inversely correlated with lifespan. Autopsies are currently being performed in an attempt to determine whether the presence of chronic pain influences cause of death.

**Speaker 2**: Thomas Hadjistavropoulos, Ph.D., FCAHS, University of Regina, Psychology and Centre on Aging and Health, Regina, Saskatchewan, Canada, thomas.hadjistavropoulos@uregina.ca @URHealthPsycLab

**Speaker 2 Abstract Title**: The role of advanced technologies in pain assessment and management in dementia

**Speaker 2 Abstract**: Some experts believe that, over the course of our lifetime, the best solutions to the problems of Alzheimer’s Disease and other dementias will not come from the health sciences but from technology development. Advanced technologies (e.g., self-driving vehicles, home monitoring technologies that detect injurious falls and call for help) have tremendous potential in the maximization of independence and quality of life in people with dementia. This presentation will review our recent work on development and evaluation of: a) computer vision technologies designed to detect and monitor pain expressions in older adults with dementia and limited ability to communicate; b) apps designed to help long-term care staff monitor pain in residents; c) interactive web-based pain education for long-term care staff working in rural and remote areas; and d) applications of social media in pain knowledge dissemination.

**Speaker 3**: Mary-Ann Fitzcharles, MD, FRCP (UK), McGill University, Faculty of Medicine, Montreal, Quebec, Canada, Mary-Ann.Fitzcharles.med@ssss.gouv.qc.ca

**Speaker 3 Abstract Title**: My grandma needs medical cannabis for her pain

**Speaker 3 Abstract**: The worldwide prevalence of chronic pain in older adults is estimated to be 25-85%, a staggering number. Medication treatment options for older adults are fraught with challenges. To name just a few, attention must be given to comorbid illnesses, drug-drug interactions and effect on mobility and cognition. It is thus understandable that patients and families may be turning to medical cannabis, commonly seen as a natural product with less harmful effects than many traditional medications. The gold standard for understanding of any medication effect is reliant on evidence accrued from randomized controlled trials (RCTs). This traditional evidence is lacking for medical cannabis in general, and especially in older adults. It is therefore necessary to turn to real world experience that will include cohort and open label studies to best understand the effects of medical cannabis in older adults. With this information the healthcare community will be better informed and better able to provide safe and competent care for this patient population. Real-world pragmatic suggestions will provide guidance when there is consideration of medical cannabis use in these patients. Empathetic patient care will be emphasized with the premise of “do no harm”.

**Learning Objective 1**: To describe new findings, from animal studies, on the differential effects of pain on lifespan as a function of sex.

**Learning Objective 2**: To recognize an update on recent developments in and outcomes of advanced technologies (e.g., computer vision) in the pain assessment of people with severe dementia and limited ability to communicate.

**Learning Objective 3**: To reflect on research and clinical guidance on the use of cannabis for pain management in older adults.

**From risk to sustainability: The evolution of transitional pain services across the lifespan**

Joel Katz^a^, Brittany N. Rosenbloom^b^, Salima S. J. Ladak^c^ and Kathryn A. Birnie^d^

^a^Psychology, York University, Toronto, Ontario, Canada; ^b^The Hospital for Sick Children, Toronto, Ontario, Canada; ^c^Toronto General Hospital & Lawrence S. Bloomberg Faculty of Nursing, Department of Anesthesia and Pain Management, Toronto, Ontario, Canada; ^d^Department of Anesthesiology, Perioperative, and Pain Medicine, University of Calgary; Department of Community Health Sciences, Calgary, Ontario, Canada

**Symposium Chair**: Joel Katz, PhD, CPsych, York University, Psychology, Toronto, Ontario, Canada, jkatz@yorku.ca

**Symposium Abstract:** Health Canada’s Canadian Pain Task Force established the prevention and management of chronic pain as a top priority for Canada. It is well known that a high proportion of individuals receiving surgical intervention go on to develop chronic postsurgical pain. Research has identified several risk factors for the transition from acute to chronic postsurgical pain in adults, however, this is a budding area of research for children and adolescents. This symposium will first discuss new research highlighting the psychosocial risk factors for the development of chronic postsurgical pain and identify areas for intervention. Identifying risk factors for chronic postsurgical pain is critical as it provides target areas for intervention. Second, this symposium will examine how The Transitional Pain Service at Toronto General Hospital has developed over time to secure sustainable multidisciplinary intervention for the prevention and management of chronic postsurgical pain. It will also discuss the evolution of the program towards opioid harm reduction. Third, this symposium will discuss the co-design of a “transitional pain service” for pediatrics. In partnering with youth, families, healthcare professionals, and health systems administrators the development of a sustainable patient-centered service is possible. Taken together, this symposium discusses the key ingredients for the prevention and management of chronic postsurgical pain across the lifespan.

**Speaker 1**: Brittany N. Rosenbloom, PhD, The Hospital for Sick Children, Toronto, Ontario, Canada, brittany.rosenbloom@sickkids.ca @BNRosenblm

**Speaker 1 Abstract Title**: Psychosocial risk factors for pediatric chronic postsurgical pain: Targets for intervention

**Speaker 1 Abstract**: Approximately 20% of youth develop chronic post-surgical pain (CPSP) that is associated with pain-related distress and co-morbid mental health outcomes, such as anxiety and depression. Identifying risk/ protective factors for the development of CPSP in youth is at its infancy. This session examines youth and parent risk/protective factors associated with the development and maintenance of pediatric CPSP, including functional limitations. Dr. Rosenbloom will share data from a large sample of youth aged 8 to 17 years undergoing major orthopedic or general surgery and their parents (n = 264). These youths and their parents completed questionnaires at four time points over the course of 12 months (pre-surgery, in-hospital, 6- and 12-months after surgery). She will discuss youth-related factors, such as presurgical general functional limitations and anxiety, as well as parent factors, such as anxiety sensitivity and anxiety, associated with the development and maintenance of CPSP. She will also discuss differences between predictors of 12-month pain-specific functional limitations as compared to general functional limitations. The findings discussed support the use of a combined diathesis-stress and interpersonal fear-avoidance model of pain to understand the transition from acute to chronic pain in the pediatric surgical population. The results of these studies also identify areas for intervention and future research.

**Speaker 2**: Salima S. J. Ladak, BScN, MN (NP), PhD, Toronto General Hospital & Lawrence S. Bloomberg Faculty of Nursing, Department of Anesthesia and Pain Management, Toronto, Ontario, Canada, salima.ladak@uhn.ca

**Speaker 2 Abstract Title**: Transitional pain program: Sustainability, evolution and lessons learned

**Speaker 2 Abstract**: Established in 2014, the Toronto General Hospital Transitional Pain Service is now a routine part of the Organization’s pre-operative, peri-operative and post-discharge pathways. This session reviews the essential success factors in program sustainability, growth and development across 3 levels. These include the individual or patient, organizational level and community level. At the individual patient level, program awareness through the pre-operative anesthesia assessment clinics as well as ward-based awareness initiatives have remained a core mechanism of patient identification. Pre-operative assessments have helped identify patients requiring customized pain management through the peri-operative period. Customized pain care following discharge, which focuses on modifiable outcomes such psychological factors and functional goals have been key to patient success. At the organizational level, awareness created among the surgical services divisions and formal linkages to health care disciplines has helped to link patients to this service. At this level, the Program is increasingly addressing needs in the practice of harm reduction. The individual level factors have uncovered for our program patients who may be at risk for substance use disorder and are identified earlier. We will describe ways in which harm reduction and clinical capacity building is taking place to address this. Our challenge remains the community-based level, which continues to provide a sub-group of patients for longer term care – beyond the first 3 post-operative months. Our team-based model including anesthesiologists, nurse practitioners, psychologists and physiotherapists has been the single most critical factor to deliver comprehensive, customized, and patient focused services.

**Speaker 3**: Kathryn A. Birnie, PhD, RPsych, University of Calgary, Department of Anesthesiology, Perioperative, and Pain Medicine; Department of Community Health Sciences, Calgary, Ontario, Canada, kathryn.birnie@ucalgary.ca @katebirnie

**Speaker 3 Abstract Title**: Co-designing health services to prevent pediatric chronic postsurgical pain

**Speaker 3 Abstract**: Preventing the transition from acute to chronic pain is the top priority identified by youth, families, and healthcare professionals for pediatric chronic pain research. Given that about 20% of children who undergo surgery will develop CPSP, ensuring effective pain management for surgery presents an ideal opportunity to stop chronic pain before it starts. “Transitional Pain Services” (TPS) have emerged in adult tertiary care as an innovative and effective health service model to prevent CPSP, but evidence for how to optimize perioperative care to prevent CPSP in children is lacking. Dr. Birnie will share phases of a human-centered design project to co-design pediatric TPS funded by a CPS Early Career Investigator Grant. She will discuss a) a survey of 85 healthcare professionals that revealed significant differences between current pediatric surgical pain management and published clinical practice guidelines, as well as health system readiness for change in pediatric perioperative care at healthcare institutions in Canada; b) interviews with youth with CPSP, parents, and healthcare professionals revealing needs, gaps, and opportunities relevant to pediatric TPS design and implementation; and c) an interactive health service blue print for pediatric TPS created during two virtual design thinking workshops, as well as key outcomes for service evaluation according to 5 youth with CPSP, 6 parents, 9 healthcare professionals, and 6 health systems administrators.

**Learning Objective 1**: Attendees will be able to identify risk factors associated with the development of chronic postsurgical pain.

**Learning Objective 2**: Attendees will be able to describe key ingredients necessary for the development of an effective and sustainable transitional pain service.

**Learning Objective 3**: Attendees will be able to consider how partnering with patients and their families, healthcare professionals, and health systems administrators contributes to user-centered design of transitional pain services.

**A Nobel therapeutic target -TRPV1-expressing sensory neurons**

Christophe Altier^a^, Feng Wang^b^ and Tomoko Ohyama^c^

^a^Snyder Institute for Chronic Diseases, University of Calgary, Calgary, Alberta, Canada; ^b^CERVO Brain Research Centre, Québec Mental Health Institute, Laval University, Quebec City, Quebec, Canada; ^c^Quantitative Life Sciences, Department of Biology, McGill University, Montreal, Quebec, Canada

**Symposium Chair**: Feng Wang, PhD, CERVO Brain Research Centre, Québec Mental Health Institute, Laval University, Quebec City, Quebec, Canada, feng.wang.1@ulaval.ca

**Symposium Abstract:** This year’s Nobel Prize in Physiology or Medicine was awarded to David Julius and Ardem Patapoutian for their discoveries of heat receptor (TRPV1) and touch receptor (Piezos), respectively. It reflects the exceptional achievement in the field of signal transduction in somatosensory system from the recent two decades. However, how the peripheral afferent information is presented and altered under different conditions still remains unclear. This symposium will cover a wide range of topics centered in nociceptors, including TRPV1-expressing sensory neurons. Speakers will address regulation of TRPV1-expressing neurons in chronic diseases, new insights of the function of TRPV1-expressing neurons, and how painful experiences during the early stages of development induces plasticity change of nociceptors and alters behavior in adulthood.

**Speaker 1**: Christophe Altier, PhD, Snyder Institute for Chronic Diseases, University of Calgary, Calgary, Alberta, Canada, altier@ucalgary.ca

**Speaker 1 Abstract Title**: New insights on the role of TRPV1 nociceptors in persistent pain

**Speaker 1 Abstract**: Sensory neurons that express the TRPV1 channel detect and transduce a variety of noxious stimuli. Sensitization of these nociceptors can lead to persistent pain in response to infection, inflammation, or injury. Identifying the mechanisms of sensitization has been key to defining the maladaptive long-lasting changes in nociceptive circuits which can precipitate the transition to chronic pain. Despite characterization of a large number of inflammatory mediators, their receptors and downstream signaling pathways, very few of these targets have led to new treatments for pain relief. Using a novel TRPV1 reporter mouse to investigate differentially expressed genes in nociceptors, we identified a previously unreported biomarker of inflammation-induced nociceptor sensitization. We examined its pronociceptive properties and tested the analgesic efficacy of clinically available inhibitors of its receptor signaling in mouse models of chronic pain. In the second part of my talk, I will present how, using chemogenetics, we demonstrated that silencing TRPV1-expressing visceral afferent neurons prevented spinal gliosis and subsequent visceral hypersensitivity (VHS) in a mouse model of colitis. In contrast, chemogenetic activation, in the absence of colitis, enhanced microglial activation associated with VHS. Our data demonstrated that activity of TRPV1 visceral afferents drive VHS through the microglial P2RY12. Targeting spinal P2RY12 signaling could be harnessed to relieve pain in IBD (Inflammatory Bowel Disease) patients who are in remission.

**Speaker 2**: Feng Wang, PhD, CERVO Brain Research Centre, Québec Mental Health Institute, Laval University, Quebec City, Quebec, Canada, feng.wang.1@ulaval.ca

**Speaker 2 Abstract Title**: Thermal and mechanical modalities converge in the noxious range

**Speaker 2 Abstract**: There are a few competing theories about how pain sensation arise from noxious stimuli, and labelled line theory has received most supports from behavioral approaches. Labelled line theory holds that distinct types of sensory neurons and neural circuits mediate different types of pain sensation. In mice, TRPV1^+^ sensory afferents were suggested as a labeled pathway for noxious heat, but not noxious mechanical sensation. However, their physiological sensitivity remains largely unknown. Using in-vivo Ca^2+^ imaging we found that most TRPV1^+^ neurons responded to heating, but not cooling stimuli. Although TRPV1^+^ neurons did not respond to innocuous brush stimulation, surprisingly, around half of them were sensitive to noxious mechanical stimulation. On the other hand, Mrgprd^+^ neurons, a subpopulation of nociceptors different from TRPV1^+^ neurons, were also sensitive to both noxious mechanical and thermal stimuli. Specifically inhibiting TRPV1^+^ neurons by using pharmacology or Mrgprd^+^ neurons with chemogenetics can inhibit both thermal and mechanical nociception, respectively. Thus, our data proved that around half of TRPV1^+^ neurons and Mrgprd^+^ neurons are polymodal nociceptors and involved in both thermal and mechanical nociception, indicating that thermal and mechanical modalities converge in the noxious range.

**Speaker 3**: Tomoko Ohyama, PhD, Quantitative Life Sciences, McGill University, Department of Biology, Montreal, Quebec, Canada, Tomoko.ohyampa@mcgill.ca

**Speaker 3 Abstract Title**: Neurocircuit mechanisms underlying the plasticity changes by nociceptive experience during development in Drosophila larvae

**Speaker 3 Abstract**: Painful (nociceptive) experiences during the early stages of development are likely to alter behavior in adulthood. However, the mechanisms that mediate such behavioral alterations, which may include changes in the response properties of neurons, in particular sensory neurons (but perhaps central and motor neurons as well), remain unclear. Here we use the fruit fly larva as a model system to address this question, given the abundance of optogenetic tools that are available to manipulate the activity of its well-characterized nociceptive sensory neurons during development. We found that intense optogenetic stimulation of nociceptive neurons in early-stage larvae induced sensitization of escape behavior in late-stage larvae, whereas unstimulated control animals showed no such sensitization. To clarify the neural mechanisms underlying this change, we knocked down various neuromodulator receptors in nociceptive neurons and determined that the octopamine receptor was involved in the sensitization process. Furthermore, we found that activation of octopamine neurons mimicked the effects of nociceptive neuron activation during development. These data demonstrate a novel mechanism by which plastic changes specific to the sensory pathway can be induced within the nociceptive circuitry of larval Drosophila.

**Learning Objective 1**: At the end of this session, participants will be able to describe the function of TRPV1-expressing sensory neurons in physiological and pathological conditions.

**Learning Objective 2**: At the end of this session, participants will be able to appraise the current approaches to study the function of sensory neurons, including in vivo calcium imaging, genetics, optogenetics, and chemogenetics.

**Learning Objective 3**: At the end of this session, participants will be able to discuss the plastic change of pain sensation during development and the effect of painful experience at early developmental stage.

**Chronic multisystem pains in Ehlers Danlos Syndromes: Diagnostic framework, clinical characterization, and biopsychosocial model of management**

Hance Clarke^a^, Nimish Mittal^b^, Rosalind Robertson^c^ and Maxwell Slepian^d^

^a^Department of Anesthesiology and Pain Medicine, University of Toronto, Toronto, Ontario, Canada; ^b^Department of Medicine, Division of Physical Medicine and Rehabilitation, University of Toronto, Toronto, Ontario, Canada; ^c^Toronto, Ontario, Canada; ^d^York University, Toronto, Ontario, Canada

**Symposium Chair**: Hance Clarke, MD, FRCPC, PhD, University of Toronto, Department of Anesthesiology and Pain Medicine, Toronto, Ontario, Canada, hance.clarke@uhn.ca @Drhaclarke

**Symposium Abstract:** Ehlers Danlos Syndromes (EDS) is a group of hereditary connective tissue disorders that present with multisystemic issues related to collagen metabolism. Traditionally, EDS was thought to induce defective collagen, predominantly in ligaments causing joint subluxations/dislocation and chronic pains. With the advancement of literature, the gamut of systemic issues in EDS has expanded and been associated with conditions like postural orthostatic tachycardia syndrome, mast cell activation disorder, and gastrointestinal manifestations. The vast constellation of medically unexplained multisystemic pain features makes EDS challenging to diagnose and treat due to the lack of knowledge about this disorder.This symposium will center around the patient journey and resources needed to support individuals living with EDS. This session will discuss the clinical presentation and pathogenetic connection in EDS and related connective tissue disorders (e.g. Hypermobility Spectrum Disorder). Data from 1200 patients assessed at the GoodHope Ehlers Danlos Syndrome Clinic at Toronto General Hospital will be presented to highlight distinctions and similarities amongst widespread chronic pain patients with multisystemic symptom concerns with and without connective tissue dysfunction. The speakers will discuss a comprehensive humanization approach combining evidence-based interdisciplinary interventions and delivery facilitation that adds value to patients overall experience. The guest speakers include an interdisciplinary panel of patients, psychologists, and individuals with lived experience on complex multisystemic pain conditions. Each speaker will deliver a 15-minute presentation, and the chair will facilitate a 20-minute discussion/question and answer session with the delegates.

**Speaker 1**: Nimish Mittal, MBBS, MD, MSc, University of Toronto, Department of Medicine, Division of Physical Medicine and Rehabilitation, Toronto, Ontario, Canada, nimish.mittal@uhn.ca @mittalnimish

**Speaker 1 Abstract Title**: Diagnostic framework, pain characteristics and pentad connection in Ehlers Danlos Syndrome -Does it differ from other chronic pain conditions?

**Speaker 1 Abstract**: Defect in collagen function or maturation is theorized to be involved in several multisystemic pain disorders that present primarily with generalized joint hypermobility. The clinical phenotype in multisystemic pain disorders is complex and evolves over time. Quite commonly, it starts as loose clumsy joints with musculoskeletal pains and transitions to generalized progressive widespread musculoskeletal pains, pelvic pains, abdominal pains, headaches, orthostatic intolerance, fatigue, and cognitive slowness. Health professionals find persistent chronic illnesses with medically unexplained multisystemic features challenging to diagnose due to the lack of knowledge of these disorders. This leads to several years of delay in the diagnosis of these complex disorders that markedly impact the health and well-being of individuals. Patients feel discredited and develop dissatisfaction with the healthcare system.This presentation will provide an overview of the pathogenesis, and diagnostic framework in the hereditary connective tissue disorder of Ehlers Danlos Syndromes. Difference in pain characteristics and manifestations between EDS and non-EDS chronic pain conditions from the data collected at the GoodHope EDS clinic will be presented. Relevant investigations and an algorithm for interprofessional management will be discussed in light of the current evidence.

**Speaker 2**: Rosalind Robertson, BA (Journalism), Toronto, Ontario, Canada, rosalind.robertson@gmail.com

**Speaker 2 Abstract Title**: Living with multi-systemic chronic pains - Issues with my tissues

**Speaker 2 Abstract**: I have had seemingly unrelated medical issues all my life - severe GI issues, a ruptured appendix, joints popping out, bruises, migraines - the list went on. No one could put it together until I was nearly 40 and was barely able to leave the house. I got a diagnosis, but I was stranded as a patient. No one knew what Ehlers Danlos Syndrome (EDS) was or how to treat it. I still have trouble getting people to believe me - that I have constant pain, fatigue, and other issues. EDS can be “liveable,” but it requires specific and constant medical interventions and management and relies on the patient being fully engaged in their care. I became my own health program advisor and built my own team of specialists and found best practices in managing EDS through rehabilitation and physiotherapy to lead a high functioning life.

**Speaker 3**: Maxwell Slepian, Ph.D., CPsych, York University, Toronto, Ontario, Canada, Maxwell.Slepian@uhn.ca

**Speaker 3 Abstract Title**: Psychological processes and treatment in Ehlers Danlos Syndrome and Generalized Hypermobility Spectrum Disorders

**Speaker 3 Abstract**: Individuals with chronic multisystemic pain disorders face an arduous journey to diagnosis and treatment. Along this journey, many become understandably wary of interacting with mental health professionals. Yet, these individuals experience co-morbid psychological disorders at much higher rates than the general population. In addition to pain and psychological distress, the majority of individuals with EDS or joint hypermobility experience complications arising from multiple physiological systems, including autonomic dysfunction and functional gastrointestinal problems. Data from patients assessed at the GoodHope EDS Clinic at Toronto General Hospital will be presented to describe rates and nature of psychological concerns for amongst patients with multisystemic symptom concerns with and without connective tissue dysfunction. These data support a biopsychosocial model wherein repeated noxious sensory stimulation and autonomic dysfunction enhance emotional dysregulation and distress. These psychological features, in turn, contribute to the maintenance of physical symptoms. Psychological treatment is an essential component of the GoodHope EDS Clinic’s multidisciplinary care model. Development of a stepped care psychology treatment model, featuring interventions based on Acceptance and Commitment Therapy and Dialectical Behavior Therapy will be described, and the adaptation and implementation of this treatment model during the COVID-19 pandemic will be addressed.

**Learning Objective 1**: Identify the gaps in health care experienced by patients living with multisystemic pain disorder of Ehlers Danlos Syndromes.

**Learning Objective 2**: Recognize the spectrum of clinical manifestations, common co-morbidities and evidence-based chronic pain management strategies in patients with Ehlers Danlos Syndromes.

**Learning Objective 3**: Identify psychological and physical comorbidity in Ehlers Danlos Syndrome and Generalized Hypermobility Spectrum Disorder and the role of psychology in a multidisciplinary clinic for these disorders.

**Chronic pain in Canadian Veterans and their families: Prevalence, mechanisms, treatment, and lived experience**

Tom Hoppe^a^, Melanie Noel^b^, Joy MacDerrmid^c^ and Ryan and Rebekah Mitchell^d^

^a^Chair, Advisory Council for Veterans, Chronic Pain Centre of Excellence for Canadian Veterans, Veteran (Ret. Sgt), Awarded the Meritorious Service Cross and Medal of Bravery, Canadian Armed Forces, Vancouver, British Columbia, Canada; ^b^University of Calgary, Calgary, Alberta, Canada; ^c^School of Physical Therapy, Faculty of Health Sciences, Western University, ON, Canada; Roth McFarlane Hand and Upper Limb Centre, St. Joseph’s Hospital, London, ON Canada; ^d^Chronic Pain Centre of Excellence for Canadian Veterans, Hamilton, Ontario, Canada

**Symposium Chair**: Tom Hoppe, MSC, MB, CD, MA, Chair, Advisory Council for Veterans, Chronic Pain Centre of Excellence for Canadian Veterans

Veteran (Ret. Sgt), Awarded the Meritorious Service Cross and Medal of Bravery, Canadian Armed Forces, Vancouver, British Columbia, Canada, vapmanag@gmail.com

**Symposium Abstract:** Chronic pain and associated mental health issues are alarmingly prevalent in Veterans (VanDenKerkhof et al., 2015; Vun et al., 2018; Seal et al., 2007), placing their children at heightened risk for the development of pain. Nevertheless, only one empirical study to date has examined pain in offspring of Veterans (Swedean et al., 2013). Given this scarcity, there is a critical need to characterize the prevalence of pain in children of Canadian Veterans and the mechanisms underlying intergenerational risk for chronic pain. Moreover, understanding of which pain treatments work best for which Veterans, is limited, and research examining sex and gender considerations and treatment components is critically needed. Through funding from the Chronic Pain Centre of Excellence for Canadian Veterans, Drs. Melanie Noel and Jennifer Stinson have brought people with lived experience (2 Veterans, a spouse, a child) to their team to be the first to examine the prevalence and drivers of chronic pain in Veterans and their children and provide deep understanding of how pain unfolds and is expressed and responded to within Veteran families. Dr. Joy MacDermid will also present novel research examining treatment needs of Veterans and how they may differ by gender and other individual differences. Finally, the lived experiences of a Veteran (Ryan Mitchell) with chronic pain and his spouse (Rebekah Mitchell) will be woven throughout and the session will be moderated by Tom Hoppe, who in recognition of his conspicuous leadership and bravery under fire was awarded the Meritorious Service Cross and Medal of Bravery.

**Speaker 1**: Melanie Noel, PhD, RPsych, University of Calgary, Calgary, Alberta, Canada, melanie.noel@ucalgary.ca

**Speaker 1 Abstract Title**: Intergenerational transmission of chronic pain in Canadian Veterans and their children

**Speaker 1 Abstract**: The etiology of chronic pain remains a mystery and current treatments are often ineffective for the majority of youth. Given that it is a treatment-resistant and often lifelong condition, uncovering prognostic biomarkers is essential for answering the most pressing research question facing the field: How do we prevent chronic pain in children before it begins?We believe the answer lies in parental chronic pain. Chronic pain can have devastating effects on a parent’s health, and in turn, the development of their children. Emerging research demonstrates that chronic pain runs in families, due to genetic and behavioral mechanisms.Chronic pain and mental health issues are alarmingly prevalent in Veterans, likely placing their children at heightened risk for the development of pain problems. Nevertheless, only one empirical US-based study has examined pain in offspring of veterans, revealing that risk for headaches was high and tended to worsen over time, especially in younger children. Given this scarcity, there is a critical need for empirical research to characterize and establish the prevalence of pain in children of Canadian veterans and understand the mechanisms underlying the intergenerational risk for chronic pain.Using a cross-sectional sample, Dr. Noel will characterize pain and mental health (PTSD, anxiety, depression, insomnia, substance use) in Canadian veterans and their children. Using in depth qualitative data from 20 Veterans, children and spouses, she will also provide an in-depth understanding of the pain experience of veterans and their children. Reflective thematic analysis will be applied to derive key themes that emerge.

**Speaker 2**: Joy MacDerrmid, PhD, School of Physical Therapy, Faculty of Health Sciences, Western University, ON, Canada.

Roth McFarlane Hand and Upper Limb Centre, St. Joseph’s Hospital, London, ON Canada

London, Ontario, Canada, jmacderm@uwo.ca

**Speaker 2 Abstract Title**: Identifying differences in chronic pain treatment needs and responses based on sex and gender in Canadian Veterans: planning for future customized interventions

**Speaker 2 Abstract**: Objective: #1: To assess whether current research on pain management in veterans considers sex and gender differences. #2: To describe the components of the chronic pain treatment programs, and possible relationships between treatment components and outcomes. Methods: Scoping Review: We searched 5 databases for studies of active-duty military and veterans with non-cancer pain who received rehabilitation or opioid/s interventions (vs. any control group) published from January 2000 to February 2021. Review #1 used Sex and Gender Equity in Research, and Sex and Gender Methods Review guidelines to assess sex and gender reporting. Review #2 used an intervention description checklist and guide to verify an intervention mapping approach to describe the content of programs. Results: Review #1: All 21 RCTs failed to report how sex/gender were integrated in their study design, sex/gender differences in their results, or how sex/gender affected their results. Review #2: Treatment programs and potential included general treatment targets (biologic, health literacy, psychologic, social) and strategies specific to certain mental health diagnoses. Process, patient, and health outcome systems benefits were variably reported. The preliminary framework for components and mechanisms of action for a chronic pain intervention is a start point for better definition of the conceptual basis for complex interventions for chronic pain. Conclusion: There is an urgent need to consider sex/gender and define the rationale or mechanistic framework to justify the components, targeting, and emphasis of multi-modal treatment programs. Without this chronic pain treatment will remain poorly understood or targeted to veterans.

**Speaker 3**: Ryan and Rebekah Mitchell, Lived Experience, Chronic Pain Centre of Excellence for Canadian Veterans, Hamilton, Ontario, Canada, tmcg@rogers.com

**Speaker 3 Abstract Title**: Lived experiences of a Veteran with chronic pain and their spouse: How pain unfolds within the family context

**Speaker 3 Abstract**: Ryan joined the Canadian Armed Forces (CAF) at 16. He was the youngest commander with the Quick Reaction Force and served alongside the U.S. Army and other countries during the Bosnia-Herzegovina conflict. After 18 years of service and two tours, he was medically released due to injuries.After his release, Ryan battled with chronic pain and declining mental health. Between doctor’s visits, trying to make sense of his diagnoses, and raising his young family, chronic pain became a constant companion for not only Ryan, but also his wife and three children. It was at this time that Ryan’s wife, Rebekah, became the family advocate. With her support, Ryan entered an interdisciplinary pain program, through which he and his family learned lifelong coping strategies.Through their family journey of chronic pain, the Mitchells became advocates for, “Once one person serves, the whole family serves.” They now devote their time to bringing awareness to this topic and breaking down the stigma around mental health and psychoeducation for Veterans. Rebekah continues to volunteer in the Veteran community in support of her family with organizations such as Wounded Warriors Canada, the Together We Stand Foundation, and the Veterans Ombud Advisory Council.To further support the cause, the Mitchell family is participating in Dr. Melanie Noel’s intergenerational project with CPCoE. In this symposium, Ryan and Rebekah will share their firsthand experiences of how chronic pain has affected their family, and why this research is critically important for the next generation.

**Learning Objective 1**: To describe the experiences of chronic pain in Veterans and their children, its connection to mental health, and underlying mechanisms.

**Learning Objective 2**: To describe sex and gender differences in chronic pain treatment options for Veterans.

**Learning Objective 3**: To describe the lived experiences of a Veteran with chronic pain and a spouse and how pain is experienced within the family context.

**Factors and mechanisms involved in the transition from acute to chronic orofacial pain**

Ana Velly^a^, Brian Cairns^b^ and Ayushi Naik^a^

^a^Faculty of Dentistry, McGill University, Montreal, Quebec, Canada; ^b^Faculty of Pharmaceutical Sciences, University of British Columbia, Vancouver, British Columbia, Canada

**Symposium Chair**: Ana Miriam Velly, D.D.S., M.Sc., Ph.D., McGill University, Faculty of Dentistry, Montreal, Quebec, Canada, ana.velly@mcgill.ca @AnaMVelly

**Symposium Abstract:** Orofacial pain (OFP) is common (30%) and has a substantial impact on quality of life. Our symposium will focus on two chronic OFPs, temporomandibular disorders (TMDs) and persistent dentoalveolar pain disorder (PDAP). These OFPs often persist regardless of the treatment received. We will examine the transition from acute to chronic OFP, looking for risk factors and mechanisms. As stated by the National Institutes of Health (NIH), “we do not fully understand how acute progresses to chronic pain at any level, from molecular to behavioral”. This search for knowledge is also a goal for the Network for Canadian Oral Health and Research (NCOHR) Orofacial Pain Working Group (OPWG), created in 2019. This symposium is a venue for researchers and clinicians involved in studying the transition from acute to chronic pain. The symposium will open with an orientation to factors associated with the transition from acute to chronic OFP, as well as a presentation of the OPWG aims by Ana Velly. This will be followed by presentations on “Potential mechanisms of the Temporomandibular disorders” by Brian Cairns; and “The impact and cost of treatment for temporomandibular disorders” by Ayushi Naik.

**Speaker 1**: Ana Velly, D.D.S., M.Sc., Ph.D., McGill University, Faculty of Dentistry, Montreal, Quebec, Canada, ana.velly@mcgill.ca @AnaMVelly

**Speaker 1 Abstract Title**: Factors associated with the transition from acute to chronic orofacial pain

**Speaker 1 Abstract**: Temporomandibular disorders (TMD) and persistent dentoalveolar pain disorder (PDAP) are the most common types of chronic orofacial pain (OFP). TMD is a collective term used to describe musculoskeletal conditions characterized by pain in the muscles of mastication and temporomandibular joint or both, and/or associated structures. PDAP is a dentoalveolar pain associated with infections, surgery, endodontic lesions, or treatment. Our study and others found that the transition from acute to chronic OFP (i.e., TMD), as well as the persistence of chronic OFP, are unfortunately common. We conducted a critical review aimed at assessing the risk factors associated with the transition from acute to chronic painful TMD. This review found that myofascial pain and pain intensity were associated with the transition risk assessed at 6-months follow-up. In 2019, the Network for Canadian Oral Health and Research (NCOHR) Orofacial Pain Working Group emerged, composed of research groups aiming to design studies to (i) develop strategies and decision-making paradigms to prevent the transition from acute to chronic orofacial pain; (ii) assess risk factors and identify biomarkers to prevent the transition from acute to chronic orofacial pain; and (iii) assess the economic and social impacts of chronic orofacial pain and its prevention. This talk will provide an overview of the current state of knowledge on the transition from acute to chronic OFP.

**Speaker 2**: Brian Cairns, PhD, University of British Columbia, Faculty of Pharmaceutical Sciences, Vancouver, British Columbia, Canada, brian.cairns@ubc.ca

**Speaker 2 Abstract Title**: Potential mechanisms of the Temporomandibular disorders

**Speaker 2 Abstract**: A number of hypothetical mechanisms have been proposed to account for chronic joint and muscle pain in Temporomandibular disorders (TMDs) that include: referral, mechanical trauma, hypoxia, and neurogenic inflammation/neuropathy. Some studies find that local anesthesia reduces pain, what suggests that, for some patients, pain is due to a peripheral mechanism. There is also evidence that TMD patients show signs of central sensitization and a loss of endogenous analgesia. A number of neuroplastic changes are proposed to contribute to pain chronification in TMD patients. TMD pain mechanisms may be associated with changes in the chemical and physical environment of the affected tissues, and it is possible that these changes will be exploitable as future biomarkers. This talk will examine theoretical pathology underlying TMD-related pain and examine peripheral and central neurobiological changes that may contribute to the development of chronic pain in this craniofacial pain disorder.

**Speaker 3**: Ayushi Naik, BDS, M.Sc. student, McGill University, Faculty of Dentistry, Montreal, Quebec, Canada, ayushi.naik@mail.mcgill.ca @AyushiNaik13

**Speaker 3 Abstract Title**: The impact and cost of treatment for temporomandibular disorders

**Speaker 3 Abstract**: Temporomandibular disorder (TMD) is the most common chronic orofacial pain, and the second most common musculoskeletal disorder (after chronic back pain). Pain intensity is usually moderate, and clinically significant pain and/or disability (GCPS II-IV) are frequent, occurring in at least 20% of the patients. Patients also report social impacts: staying at home more than usual and taking time off work. The most used reversible treatments include patient education with self-care, intra-oral appliances, and pharmacological pain control. A study in England estimates that the 6-month period total cost per person varied from £321 to £519 (CAD 546 to CAD 883), where the major contributor was consultation. Significant pain and disability predicted an increased healthcare cost. The direct out of-pocket cost averaged £334 (CAD 568) per person per 6-month period. The greatest impact of TMD on indirect cost was related to reduced productivity (e.g., problems with concentration), which averaged £905 (CAD 1539) per person. We are conducting a systematic review aimed at putting in evidence the cost outcomes (cost-effectiveness, cost-utility, and cost-benefit analyses) of the most common treatments used to manage TMD. Knowing the cost involved in treating TMD is significant, as it will help develop more effective and efficient health care pathways and policies for patients with TMD. This talk will provide an outline of the existing knowledge on the impact and cost of TMD.

**Learning Objective 1**: Examine the potential risk factors implicated in the transition from acute to chronic orofacial pain.

**Learning Objective 2**: Examine theoretical pathology underlying TMD and examine peripheral and central neurobiological changes that may contribute to the development of chronic pain in this craniofacial pain disorder.

**Learning Objective 3**: Review the impact and cost of temporomandibular disorders.

